# Biomachining in manufacturing: mechanisms, processes, applications and future perspectives

**DOI:** 10.1186/s40643-026-01143-z

**Published:** 2026-10-13

**Authors:** Jingyu Song, Hui Huang, Weibin Shi

**Affiliations:** 1 Institute of Manufacturing Engineering, Huaqiao University, No. 668 Jimei Avenue, Jimei District, Xiamen, 361021 Fujian Province China; 2 College of Mechanical Engineering and Automation, Huaqiao University, No. 668 Jimei Avenue, Jimei District, Xiamen, 361021 Fujian Province China; 3 State Key Laboratory of High-Performance Tools, Huaqiao University, No. 668 Jimei Avenue, Jimei District, Xiamen, 361021 Fujian Province China

**Keywords:** Biomachining, Material removal mechanisms, Bioelectrochemical dissolution, Hybrid biomachining, Microfabrication, Process optimization

## Abstract

Biomachining (BM) is an emerging bio-assisted manufacturing approach that uses microbial metabolism and bioelectrochemical reactions to remove material under mild conditions. Due to its low mechanical loading and limited thermal effects, BM has potential in low-damage microfabrication, surface functionalization, microfluidic mold fabrication, abrasive tool dressing, and functional device modification. This review critically summarizes BM with respect to removal mechanisms, process technologies, modeling and optimization strategies, manufacturing applications, and engineering challenges. The biological and electrochemical mechanisms of BM are discussed first, followed by conventional and hybrid BM processes and their comparison with conventional and non-conventional machining technologies. Current applications are reviewed with emphasis on the gap between laboratory demonstrations and scalable industrial implementation. Major challenges include low removal efficiency, limited dimensional accuracy, unstable microbial activity, weak process controllability, contamination risk, waste treatment, and biosafety management. Future development should focus on standardized evaluation, microbial stability control, mechanism-data fusion modeling, hybrid process intensification, and dedicated equipment. Machine learning-assisted prediction, Internet of Things-enabled monitoring, digital twin-based closed-loop control, and sustainability/techno-economic validation are also important for moving BM toward controllable and scalable manufacturing.

**Graphical abstract:**

**Figure:**
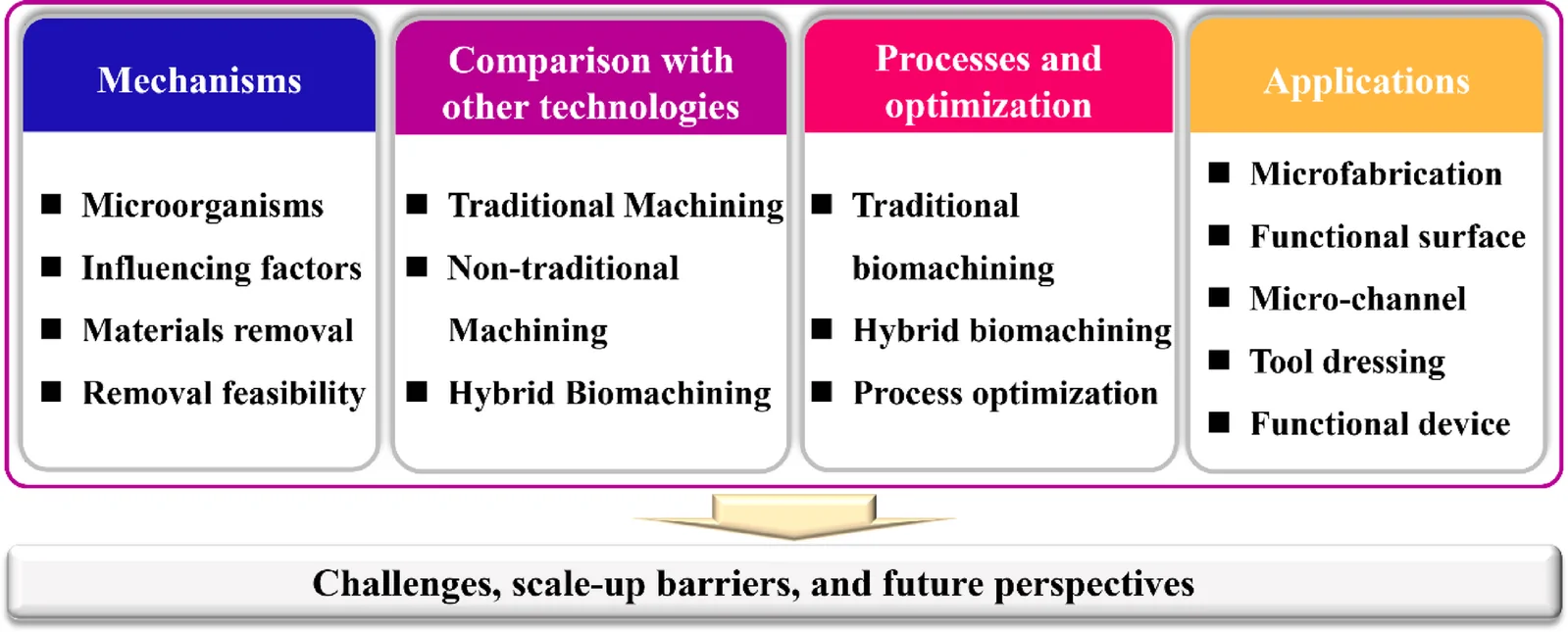

**Supplementary Information:**

The online version contains supplementary material available at https://doi.org/10.1186/s40643-026-01143-z.

## Introduction

### Background of advanced manufacturing

With the rapid development of electronic information, advanced healthcare, energy, and environmental sectors, the demand for microscale manufacturing, functional surface engineering, and high-performance components has continued to increase (Liu et al. 2026a; Ince and Özel 2025; Song et al. 2023; Farooq et al. 2022). To meet the growing requirements of precision micromachining, non-conventional machining methods, including chemical machining, laser machining, and electrical discharge machining, have been widely developed (Revilla et al. 2025; Kuriachen 2024; Zhu et al. 2024). However, these technologies still exhibit limitations in machining costs and energy consumption (Shokrani et al. 2024; Hocheng et al. 2012b). In this context, sustainable and low-carbon manufacturing requires alternative machining approaches that are lower-cost, consume less energy, and cause minimal workpiece damage, thereby promoting the development of emerging technologies such as biomachining (BM).

### Emergence and evolution of biomachining

Bioleaching, Microbiologically Influenced Corrosion (MIC), and BM all involve microorganism-mediated dissolution of materials (Knisz et al. 2023; Tezyapar Kara et al. 2023). These processes differ substantially in their engineering purposes and process controls. Bioleaching aims to recover valuable components from minerals, tailings, and waste metals and is generally regarded as a resource-extraction-oriented metallurgical process that focuses on improving metal dissolution efficiency (Elander et al. 2025). MIC refers to the accelerated corrosion and degradation of materials caused by microbial activity, with emphasis on corrosion failure mechanisms and protection strategies (Catherine et al. 2026). In contrast, BM is a manufacturing-oriented process that utilizes microbial metabolic characteristics to achieve controlled material removal. These characteristics include the transformation of highly oxidizing ions, secretion of catalytic enzymes, and formation of localized removal environments. Therefore, BM can be used to fabricate designed geometries, microstructures, and functional surface features, showing potential for precision micromachining (Hocheng and Jadhav 2015; Ting et al. 2000). In 1996, Uno et al. (1996) first used *Acidithiobacillus ferrooxidans* to fabricate microgrooves on copper and proposed the concept of BM.

### Research progress and historical development

A literature search was conducted using Web of Science, Scopus, and Google Scholar for publications from January 1996 to March 2026. The main keywords included ‘biomachining’, ‘microbial machining’, ‘biological machining’, and ‘bio-etching’. To improve transparency, a systematic literature search and selection workflow was established, as shown in Fig. S1. Studies were included if they addressed BM mechanisms, microorganisms, culture conditions, material removal processes, process parameters, hybrid biomachining (HBM), modeling and optimization, manufacturing applications, sustainability, or future development. Studies related only to bioleaching, biomining, microbial corrosion, bioremediation, or general microbiology were excluded if they did not involve manufacturing-oriented material removal or BM applications.

**Fig. 1:**
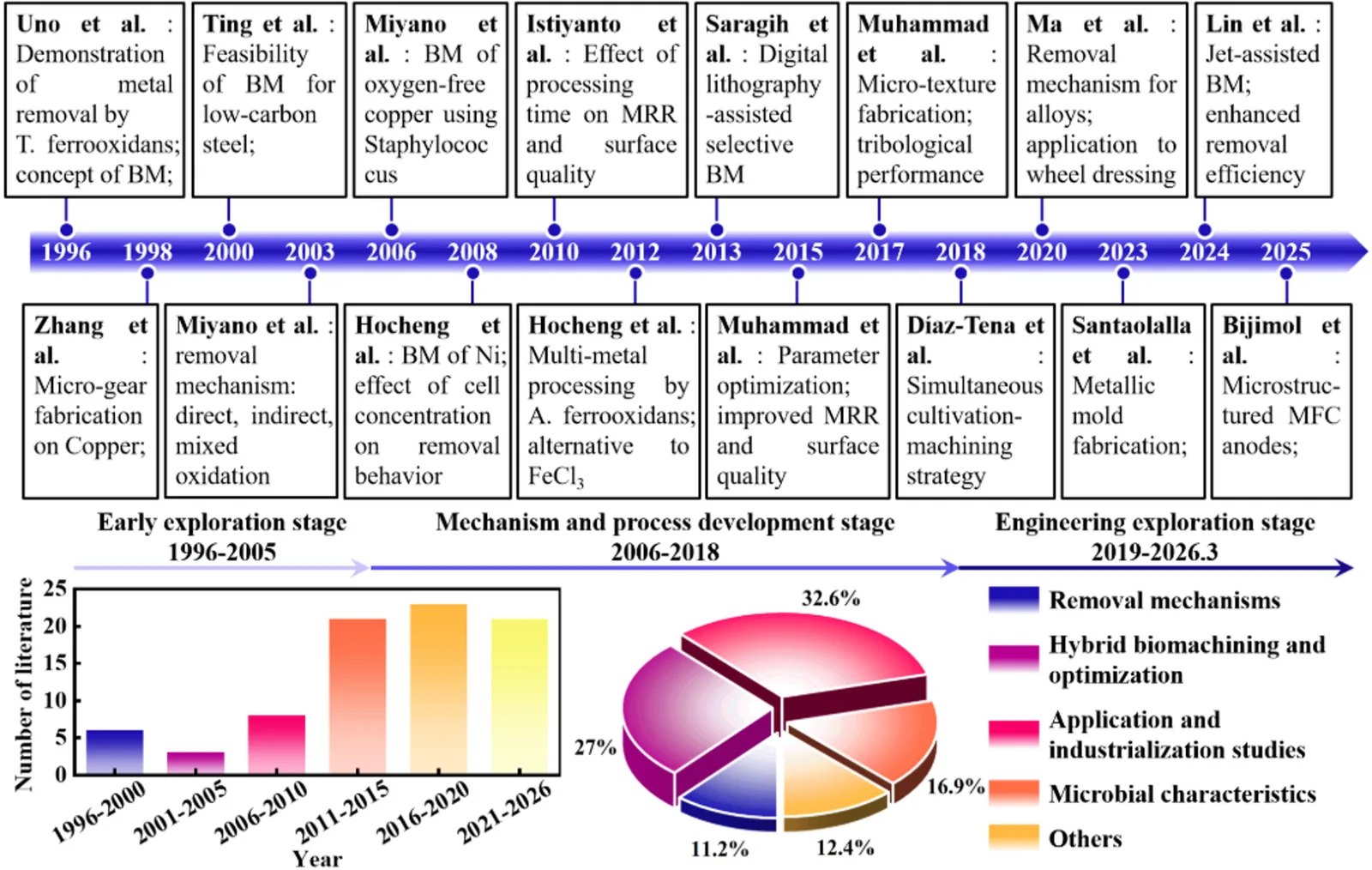
Evolution of biomachining: development history, literature analysis, and research hotspots. Created by the authors based on the literature survey. *Note:* BM, biomachining; MRR, material removal rate; MFC, microbial fuel cell

As shown in Fig. 1, the development of BM can be summarized into three progressive stages. The early exploration stage from 1996 to 2005 demonstrated the feasibility of using microorganisms as material removal agents by revealing the roles of microbial oxidation and metabolic products in metal dissolution (Kikuchi et al. 1999; Uno et al. 1996). The mechanism and process development stage from 2006 to 2018 shifted the focus toward microorganism selection, material compatibility, and process parameter regulation to improve material removal rate (MRR), surface roughness (SR), and controllability (Muhammad et al. 2015; Díaz-Tena et al. 2014). The engineering exploration stage from 2019 to 2026 introduced external energy fields, hybrid strategies, and optimization methods to overcome the limitations of conventional BM and expand its laboratory-scale applications in microstructure fabrication and functional surface preparation (Bijimol et al. 2025; Ma et al. 2025a; Santaolalla et al. 2023).

Overall, BM has evolved from feasibility demonstration toward mechanism clarification, process regulation, and engineering exploration. Its low mechanical loading, limited thermal damage, and potential environmental advantages have attracted increasing attention in microfabrication, surface engineering, and tool conditioning (Pradeep et al. 2024; Eskandarian et al. 2013; Hocheng et al. 2012a, b, c; Liu et al. 2009). However, the boundaries of its practical application remain unclear.

### Aim and structure of this review

Several review articles have summarized BM from different perspectives, including microbial metal etching, process principles, operating parameters, micromachining feasibility, and manufacturing applications. However, most existing reviews focus on selected aspects of BM. At the same time, an integrated framework linking microbial metabolism, electrochemical reactions, mass transfer, material removal mechanisms, hybrid processes, process modeling, application maturity, sustainability, and industrial scale-up remains insufficient. As summarized in Table 1, previous reviews have provided valuable foundations, but further integration from fundamental mechanisms to controllable manufacturing is still needed.

**Table 1:** Comparison of previous reviews on biomachining

Review	Main focus	Main coverage	Remaining gaps
Hocheng and Jadhav (2015)	BM as a micromachining process	Microorganisms, process principles, MRR, surface integrity, and microscale features	Limited discussion of HBM, modeling, sustainability, and scale-up
Díaz-Tena et al. (2016)	Metal etching via microorganisms	Direct/indirect mechanisms, microorganisms, operating parameters, MRR, and SR	Limited discussion of process modeling, optimization, HBM, application maturity, and industrial process control
Vigneshwaran et al. (2018)	BM for metals	Mechanisms, BM procedure, FeSO_4_ concentration, temperature, and MRR	Limited discussion of applications, surface integrity, and modeling
Ma et al. (2019)	State overview and technology analysis	Microorganisms, culture media, mechanisms, materials, and application limitations	Limited recent progress on process optimization, sustainability, and application
Ma et al. (2025b)	Application status and prospects in manufacturing	Manufacturing applications, application-related technologies, effects, hindering factors, and future directions	Less emphasis on thermodynamic/kinetic analysis, biological stability, and modeling
Suresh et al. (2025)	Sustainable alternative to conventional micromachining	micromachining comparison, mechanisms, culture conditions, parameters, applications, and future directions	Limited discussion of ML/IoT, digital twins, LCA, and application maturity

Compiled by the authors based on the cited literature

BM, Biomachining; MRR, Material removal rate; HBM, Hybrid biomachining; SR, Surface roughness; ML, Machine learning; IoT, Internet of things; LCA, Life cycle assessment

To address these gaps, this review aims to provide an integrated and manufacturing-oriented overview of BM. Compared with previous reviews, this work provides a more integrated and manufacturing-oriented perspective. It links microbial metabolism, electrochemical reactions, mass transfer, and material removal with process comparison, HBM, process modeling, intelligent optimization, application maturity, and future routes for sustainable and controllable BM.

**Fig. 2:**
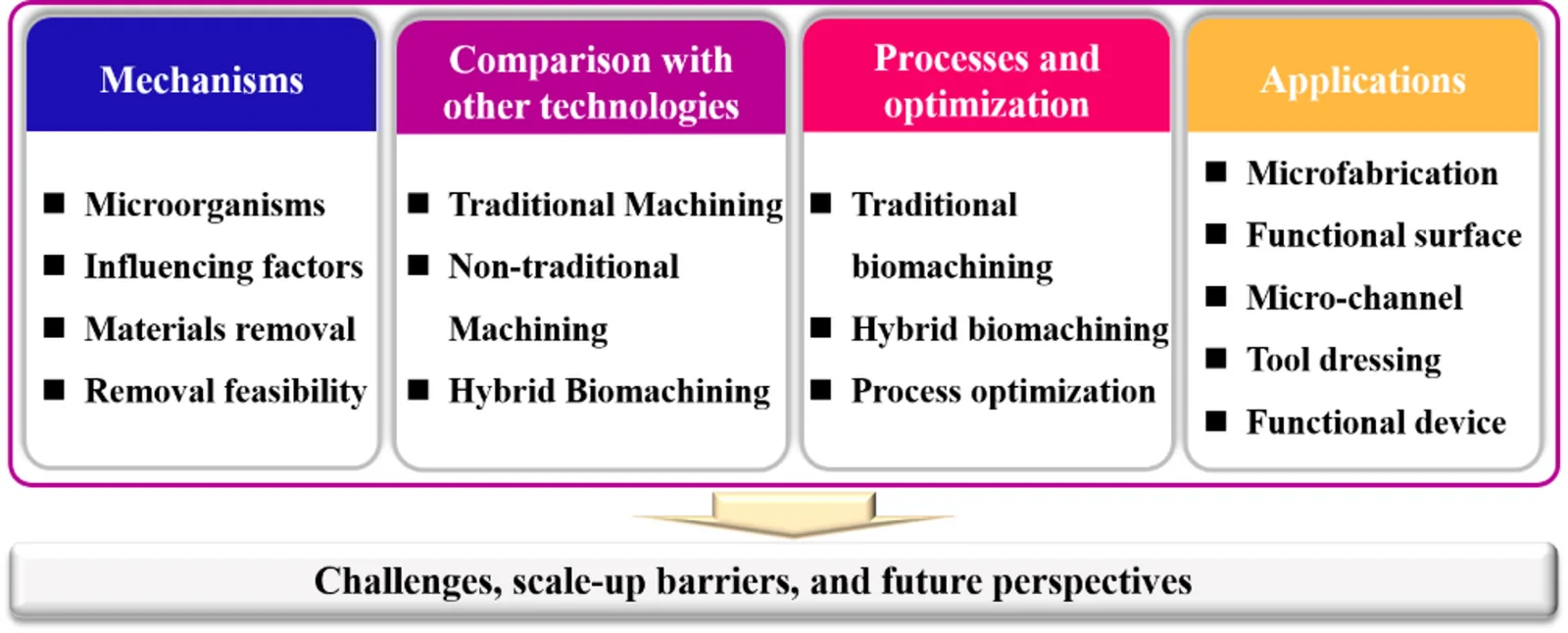
Framework and research content of the review. Created by the authors

As shown in Fig. 2, this review is organized around five aspects: mechanism analysis, process comparison, BM technologies, manufacturing applications, and future development. It discusses biological and electrochemical mechanisms, compares BM with other machining technologies, reviews conventional and hybrid BM processes, summarizes manufacturing applications, and identifies scientific barriers and future development routes.

## Mechanisms of biomachining

The interaction among microbial metabolism, solution chemistry, interfacial electrochemical reactions, and material dissolution controls BM. Understanding these mechanisms is essential for explaining material removal behavior and improving process controllability. This section summarizes the representative microorganisms used in BM, the key factors affecting microbial activity and material removal, and the direct, indirect, and hybrid removal mechanisms. Thermodynamic and kinetic assessments are further used to evaluate the feasibility of material removal and the reaction-rate characteristics. This mechanistic foundation supports the subsequent comparison with other machining technologies and the development of controllable BM processes.

### Microorganisms used in biomachining

Microbial metabolic activity determines material removal capability and process stability. Different strains promote material dissolution mainly through Fe^2+^ oxidation, organic acid, or the production of oxidative metabolites (Ma et al. 2025b). Representative microorganisms and their culture conditions are summarized in Table 2. Among them, *Acidithiobacillus ferrooxidans* and *Acidithiobacillus thiooxidans* are currently the most widely used strains, as they promote metal dissolution primarily through the Fe^2+^ oxidation cycle (Suresh et al. 2025). *Aspergillus niger* mainly facilitates material removal through organic acids and glucose oxidase (Zhang et al. 2020; Jadhav and Hocheng 2015, 2014; Eskandarian et al. 2013). *Staphylococcus* sp. can create acidic or alkaline corrosive microenvironments through different metabolic pathways, thereby enabling localized material dissolution (Matlani and Kadam 2021; Shikata et al. 2009; Kikuchi et al. 1999). In addition, strains such as *Paracoccus versutus*, *Leptospirillum ferrooxidans*, and *Sulfobacillus thermosulfidooxidans* have also been applied to BM of different material systems (Pradeep et al. 2024; Díaz-Tena et al. 2018, 2016; Hoque and Philip 2011).

**Table 2:** Representative microorganisms used in biomachining and related culture conditions

Microorganism	Typical materials	Metabolite	MRR	Limitation	References
*Acidithiobacillus ferrooxidans*	Cu, Ni, Al, Fe, Co, carbon steel, Cu alloy, Nitinol	Oxidizes Fe^2+^ to Fe^3+^; promotes oxidative dissolution	Cu: 23.2 mg/ (cm^2^∙h); Cu: 20 μm/h	Requires strong acidic conditions; slow growth;	Kohn et al. (2024); Verma et al. (2018); Jhajj et al. (2018)
*Acidithiobacillus thiooxidans*	Cu, Al, Zn, Mg, Mo, carbon steel, brass, Al alloy	Oxidizes sulfur compounds and contributes to acidic dissolution	Cu: 22.2 mg/h; Ni: 1.8 mg/h	Sulfur oxidation depends on the sulfur source	Hocheng et al. (2014); Kurosaki et al. (2003); Kumada et al. (2001)
*Aspergillus niger*	Cu, Sn, Ni, Al, Al alloy	Produces organic acids and glucose oxidase	Cu: 11 mg/h	Organic acid production varies with culture conditions	Zhang et al. (2020); Jadhav and Hocheng (2014); Eskandarian et al. (2013)
*Staphylococcus* sp.	Cu, Brass	Produces lactic acid and ammonia	Cu: 11.9 μm/h	limited controllability for precision machining	Matlani and Kadam (2021); Shikata et al. (2009); Miyano et al. (2006)

Compiled by the authors based on the cited literature

Unlike conventional machining, the material removal capability of BM depends not only on the species of microorganism but also on cell density, metabolic activity, and culture conditions. Microbial populations usually undergo lag, exponential, and decline phases, during which cell number and metabolic rate change with processing time, thereby affecting MRR and process stability (Singh et al. 2019). To maintain microbial activity and support continuous oxidant regeneration, recent studies have explored strain preservation, immobilized cultivation, online cultivation, and culture medium regulation (Song et al. 2025; Santaolalla et al. 2021, 2020; Díaz-Tena et al. 2018, 2017b). Although current strategies have improved microbial activity and stability, they still rely largely on empirical optimization.

In addition to culture regulation, biological stability is critical for BM. During long-term cultivation or repeated processing, genetic instability, mutation, metabolic variability, biofilm evolution, microbial competition, and contamination may change metabolite production, Fe^2+^/Fe^3+^ regeneration, cell adhesion, and material removal behavior, thereby reducing MRR stability and process repeatability. The key barrier is the lack of quantitative relationships among microbial metabolic behavior, mass transfer, material dissolution, and machining responses. Establishing these relationships is essential for stable, efficient, and scalable BM.

### Influencing factors

In addition to microorganism species, metabolic activity, and cell density, material removal behavior in BM is jointly affected by the culture environment, metabolite concentration, solution state, and intrinsic material properties, as shown in Table 3. Unlike conventional machining, BM is not controlled only by external processing parameters. The coupled effects of microbial metabolism, solution electrochemistry, and interfacial reactions at the material surface govern the material’s behavior.

**Table 3:** Factors influencing biomachining performance

Category	Factor	Main role	MRR	SR	References
Microbial factors	Microbial strain	Controls metabolite type and removal pathway	Increase	Microorganism-dependent	Pradeep et al. (2022); Díaz-Tena et al. (2016)
	Cell concentration	Enhances metabolite production	Increase	Decrease	Díaz-Tena et al. (2014); Chang et al. (2008)
Culture and Solution environment	Temperature	Regulates microbial and enzymatic activity	Initial increase, then decrease	Not reported	Singh et al. (2018)
	pH	Affects microbial growth and product precipitation	Decrease	Decrease	Santaolalla et al. (2025); Matlani and Kadam (2021)
	Dissolved oxygen	Promotes aerobic microbial activity	Significant increase	Decrease	Indianto et al. (2023)
	Nutrient composition	Regulates microbial survival and growth	Microorganism-dependent	Material-dependent	Díaz-Tena et al. (2018); Yang et al. (2009)
	Supernatant volume	Changes the reactive species availability	Increase	Not reported	Jadhav and Hocheng (2013)
	Metabolite-to-reactant ratio	Affects reaction rate and product inhibition	Increase	Not reported	Santaolalla et al. (2024)
Operating parameters	Agitation/mixing	Enhances mass transfer	Increase	Not reported	Muhammad and Ko (2014)
	Sample inclination angle	Changes surface contact and fluid retention	Slight increase	Increase then decrease	Istiyanto et al. (2014)
Substrate and surface	Metal surface condition	Affects passivation and interfacial reaction	Decrease	Increase	Ma et al. (2020a); Liu et al. (2019)

Compiled by the authors based on the cited literature

The cultural environment directly affects microbial growth and metabolic capability. Factors such as culture temperature, pH, dissolved oxygen (DO), medium composition, and shaking speed influence cell density, metabolic rate, and metabolite production (Ma and Huang 2020a). Appropriate temperature and pH help maintain microbial activity, while sufficient oxygen supply and suitable medium composition promote the metabolism of aerobic microorganisms (Jadhav and Hocheng 2014; Yang et al. 2009; Johnson et al. 2007). Previous studies have shown that changes in culture parameters can significantly alter microbial oxidation capability and MRR (Muhammad and Ko 2014). However, most studies still focus on single-factor optimization, and the coupled effects among different culture parameters and their influence on the stability of material removal remain insufficiently understood.

Metabolite concentration is another key factor affecting MRR, but its effect does not increase monotonically. When microbial metabolic activity is insufficient, Fe^3+^ regeneration decreases, leading to a gradual reduction in MRR (Santaolalla et al. 2024). Conversely, excessive iron ion concentration or unsuitable pH conditions can promote the formation of precipitates such as jarosite (KFe_3_(SO_4_)_2_(OH)_6_), which consumes dissolved Fe^3+^ and inhibits material removal (Santaolalla et al. 2025; Ma et al. 2024; Díaz-Tena et al. 2018). In addition, Cu^2+^ accumulation and reduced oxygen availability may suppress microbial activity (Muhammad et al. 2015). Therefore, metabolite concentration should be properly regulated. Simply increasing oxidant or metal-ion concentration does not necessarily lead to sustained improvements in machining efficiency.

Material composition, microstructure, and surface state further determine the selectivity of removal and interfacial reaction resistance during BM. Compared with pure metals, alloys may exhibit stronger selective dissolution due to potential differences among constituent elements and micro-galvanic effects (Ma et al. 2020a). In sintered and cast materials, microstructural heterogeneity can also affect localized corrosion and MRR (Pradeep et al. 2023). However, dense oxide films, passive layers, and adhered reaction product layers on the material surface can hinder interfacial reactions between metabolites and the substrate, thereby reducing MRR (Lin et al. 2022; Liu et al. 2019). Therefore, BM is not governed solely by microbial metabolic capability but is also strongly constrained by surface chemistry and interfacial mass-transfer behavior.

Overall, BM is a multivariable, coupled process rather than one controlled by a single parameter. Although previous studies have identified the effects of individual factors on MRR, these isolated trends may not be directly applicable under coupled microbial, chemical, and material-interface conditions. The key barrier is the lack of quantitative models linking microbial metabolism, culture environment, metabolite concentration, mass transfer, and interfacial reactions. Therefore, multivariable modeling and process control are needed to ensure a stable, controllable BM.

### Material removal mechanisms

BM of metallic materials is essentially an electrochemical dissolution process in which metal atoms lose electrons, forming soluble ions in a microorganism-mediated environment. Depending on whether microorganisms interact directly with the material surface or regulate dissolution via extracellular metabolites, three material removal mechanisms have been proposed: direct, indirect, and hybrid removal.

**Fig. 3:**
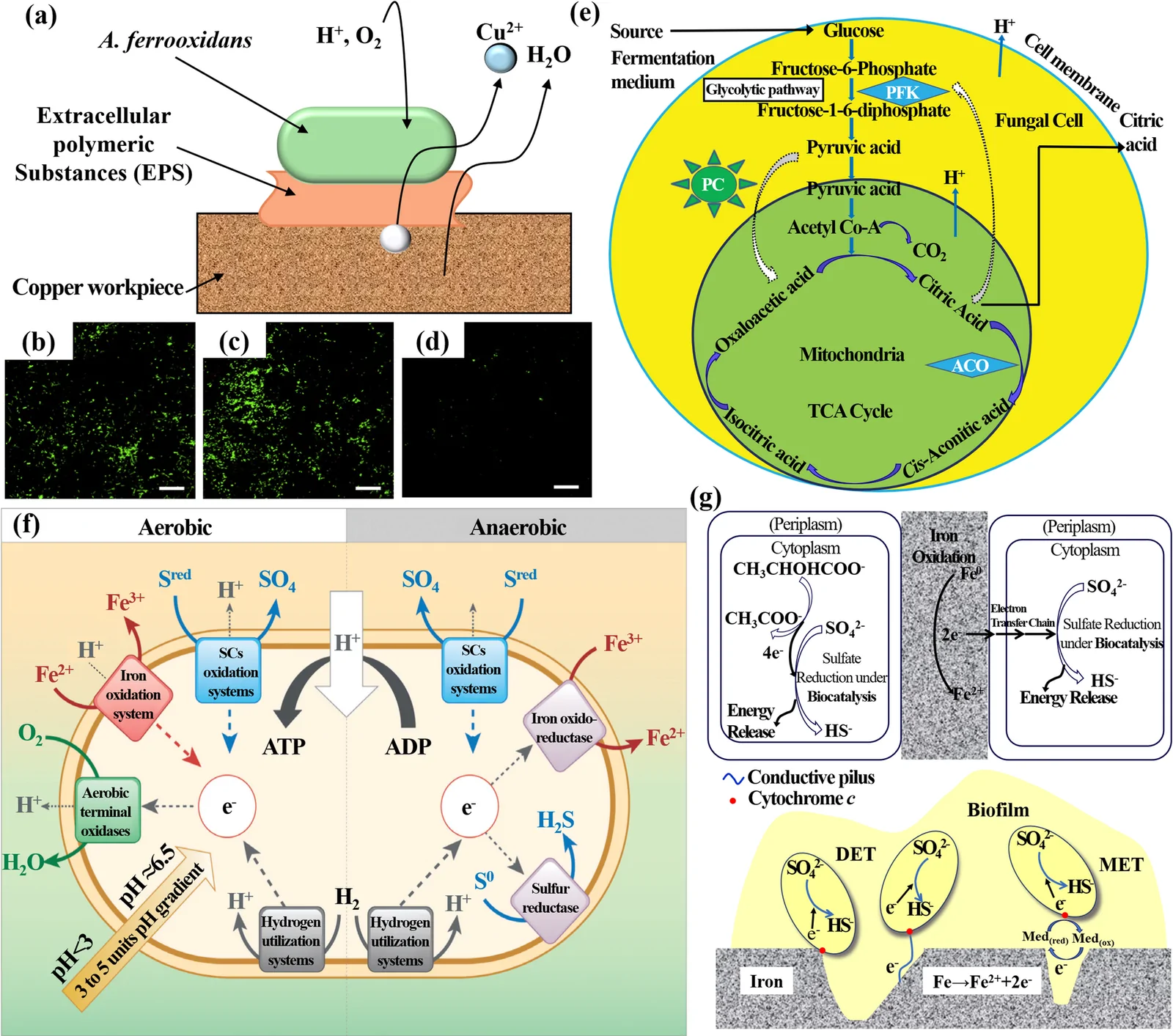
Material removal mechanisms of biomachining. **a** Direct oxidative removal by *Acidithiobacillus ferrooxidans*; microbial adhesion after **b** 1 day, **c** 7 days, and **d** 7 days followed by rinsing; **e** indirect material removal mechanism of *Aspergillus niger*; hybrid removal mechanisms of **f***Acidithiobacillus ferrooxidans* and (g) sulfate-reducing bacteria. Panel sources and permissions: **a** reproduced from Fig. 1 of Díaz-Tena et al. (2017b), reprinted by permission of the publisher (Taylor & Francis Ltd, https://www.tandfonline.com). **b**–**d** reprinted from Fig. 1 of Wang et al. (2014), Copyright (2014), with permission from Elsevier. **e** adapted from Fig. 2 of Behera (2020), reprinted by permission of the publisher (Taylor & Francis Ltd, https://www.tandfonline.com). **f** reprinted from Fig. 1 of Quatrini and Johnson (2019), Copyright (2019), with permission from Elsevier. **g** adapted from Figs. 2 and 3 of Li et al. (2018), Copyright (2018), with permission from Elsevier

As shown in Fig. 3a, the direct removal mechanism involves microbial attachment and extracellular electron transfer between cells and the metal surface (Indianto et al. 2023; Díaz-Tena et al. 2014). As shown in Fig. 3b–d, microscopic observations have confirmed microbial attachment and participation in the dissolution process (Wang et al. 2014). However, efficient biofilm formation and electron transfer are difficult to maintain during machining, which results in lower removal efficiency (Lilova et al. 2007; Zhang and Li 1998). Therefore, the contribution of the direct removal mechanism to overall material removal remains limited.

Indirect removal is the dominant material removal pathway in BM. As shown in Fig. 3e, microorganisms can generate or transform Fe^3+^, oxalic acid, and H_2_O_2_ during metabolism (Zhang et al. 2020; Jadhav and Hocheng 2014). These extracellular acidic or strongly oxidizing species etch materials through redox reactions (Jadhav and Hocheng 2016, 2015; Eskandarian et al. 2013). The dominant role of indirect removal has been supported by variations in etching efficiency under different metabolite concentrations (Muhammad et al. 2015; Xenofontos et al. 2015; Jadhav et al. 2013; Yang et al. 2009). Compared with direct removal, indirect removal offers a more controllable reaction pathway because material dissolution is primarily governed by extracellular oxidizing species rather than unstable direct contact between microbial and the material surface.

The hybrid removal mechanism is a synergistic process in which microbial surface attachment and extracellular metabolite oxidation jointly contribute to material removal. As shown in Fig. 3f and g, *Acidithiobacillus ferrooxidans* and sulfate-reducing bacteria can promote material dissolution through both extracellular electron transfer and oxidation cycles (Li et al. 2018; Istiyanto et al. 2011; Shikata et al. 2009). However, the relative contributions of direct and indirect removal vary among different microorganisms and processing conditions (Pradeep et al. 2022; Xenofontos et al. 2015). Therefore, the hybrid removal mechanism remains limited by insufficient in situ characterization and quantitative analysis of contributions, making it difficult to control material removal and predict machining quality.

Among these three mechanisms, indirect oxidation-mediated dissolution is currently regarded as the main pathway in BM, because extracellular oxidants and metabolites can continuously participate in metal dissolution without requiring stable microbial attachment. Direct removal provides fundamental evidence of microorganism-material interactions, but its contribution is often limited by low electron-transfer efficiency and insufficient biofilm stability. The hybrid mechanism combines these two pathways and shows potential for improving removal efficiency.

### Thermodynamic and kinetic assessment

**Fig. 4:**
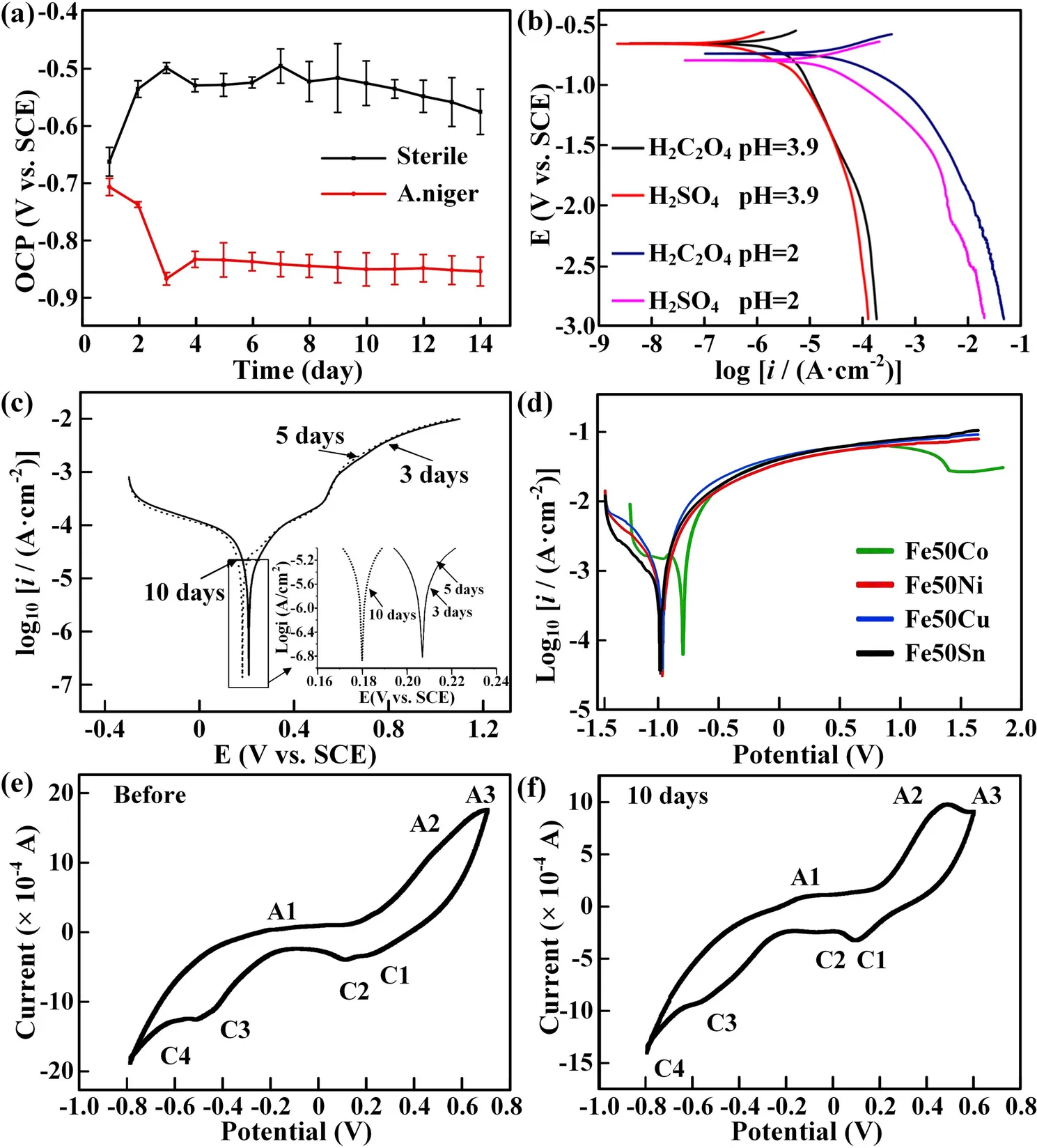
Thermodynamic and kinetic analysis of biomachining. **a** Open-circuit potential (OCP) during incubation with and without *Aspergillus niger*, measured versus a saturated calomel electrode (SCE); potentiodynamic polarization curves at **b** different pH values, **c** different machining times and **d** different material components; electrochemical impedance spectroscopy spectra **e** before BM and **f** after 10 days of BM. Panel sources and permissions: **a** and **b** reprinted from Figs. 6 and 18, respectively, of Zhang et al. (2020), Copyright (2020), with permission from Elsevier. **c**, **e** and **f** reprinted from Figs. 3, 7a, and d, respectively, of Liu et al. (2019), Copyright (2019), with permission from Elsevier. **d** reprinted from Fig. 9 of Lin et al. (2022), Copyright (2022), with permission from Elsevier

The feasibility assessment of BM has largely drawn on studies of bioleaching and MIC. Since indirect removal is generally the dominant pathway, thermodynamic and kinetic analyses can be used to evaluate the feasibility of material removal and the tendency toward dissolution (Zhang and Li 1999). Thermodynamic analysis supports material selection, prediction of preferential dissolution, and redox system design. In contrast, kinetic analysis reveals the effects of pH, oxidation–reduction potential (ORP), open circuit potential (OCP), metabolite concentration, and mass transfer on MRR, as shown in Fig. 4. However, direct removal depends on the interfacial microenvironment and microbial oxidation potential, making its key parameters difficult to quantify. Therefore, coupling thermodynamic and kinetic analyses with process parameter design is essential for developing BM from empirical regulation toward quantitative process design.

At the thermodynamic level, the Nernst equation can describe the effects of ion activity, temperature, and number of electrons transferred on the system potential (Ma et al. 2020b), as shown in Eq. (1):

1 $$ E = E^{0} {-}\left( {RT/nF} \right) \cdot \ln Q $$

where *E* is the equilibrium electrode potential (V); *E*^*0*^ is the standard electrode potential (V); *R* is the gas constant (8.31 J·mol^−1^·K^−1^); *T* is the thermodynamic temperature (K); *n* is the number of electrons transferred; *F* is the Faraday constant (96,485 C·mol^−1^); and *Q* is the reaction quotient.

The Gibbs free energy relationship can further be used to evaluate reaction spontaneity, as shown in Eq. (2):

2 $$ \Delta G = - nF \cdot \Delta E $$

where *ΔG* is the Gibbs free energy change (J·mol^−1^), and *ΔE* is the electrode potential difference between the cathodic and anodic reactions (V), which reflects the thermodynamic driving force for metal dissolution. When *ΔE* > 0 and *ΔG* < 0, metal oxidative dissolution is thermodynamically feasible (Ge and Isgor 2007). As shown in Fig. 4a, parameters such as ORP, OCP, Pourbaix diagrams, and equilibrium potential differences can be used to assess the tendency toward material removal (Zhang et al. 2020; Díaz-Tena et al. 2018). For BM systems driven by the Fe^2+^/Fe^3+^ cycle, changes in the Fe^2+^/Fe^3+^ ratio alter the redox potential of the system and thereby affect metal dissolution capability (Lin et al. 2024). Metal components with lower corrosion potentials or larger potential differences relative to the oxidant are more likely to dissolve preferentially as anodic phases (Lin et al. 2022; Ma et al. 2020a). Thus, thermodynamic analysis provides a basis for selecting machinable materials, determining pH ranges, and designing oxidant systems.

At the kinetic level, polarization curves and the Tafel relationship can be used to characterize interfacial reaction rates (Ge and Isgor 2007), as shown in Eq. (3):

3 $$ \eta = b\log_{10} \left( {i/i_{0} } \right) $$

where *η* is the overpotential (V), representing the deviation of the actual electrode potential from the equilibrium potential; *i* is the current density (A·cm^−2^); *i*_*0*_ is the exchange current density (A·cm^−2^); *b* is the Tafel slope (V· dec^−1^), reflecting the kinetic characteristics of the electrode reaction.

Current density can be further related to the metal dissolution rate through Faraday’s law, as shown in Eq. (4):

4 * r = iM/nF *

where *r* is the mass removal rate per unit area (g·cm^−2^·s^−1^); *i* is the anodic dissolution current density (A·cm^−2^); *M* is the molar mass of the metal (g·mol^−1^); *n* is the number of electrons transferred in the metal dissolution reaction; and *F* is the Faraday constant (96,485 C·mol^−1^). These relationships provide a theoretical basis for predicting MRR.

As shown in Fig. 4b–f, electrochemical impedance spectroscopy can identify limiting factors such as charge-transfer resistance, diffusion impedance, solution resistance, and interfacial film resistance. These factors affect the interfacial current response and material dissolution rate (Zhang et al. 2020; Liu et al. 2019). When material removal is controlled by charge transfer, redox species, pH, and the microbial metabolic environment should be optimized (Lin et al. 2022; Ma et al. 2020a). When mass transfer is the limiting factor, reactant transport can be enhanced through agitation, flow-field design, immobilized cultivation, or assisted energy fields.

Current thermodynamic and kinetic analyses in BM are still largely adapted from bioleaching and MIC studies, and their manufacturing-guiding capability remains limited. Thermodynamic analysis can assess the tendency of a reaction, but it cannot directly predict MRR. Kinetic models can describe interfacial reaction rates, but they often neglect microbial growth, metabolic fluctuations, biofilm evolution, and mass transfer. The key barrier is the lack of a quantitative framework linking electrochemical reactions, microbial metabolism, mass transfer, and machining responses. This limitation hinders process parameter design, online monitoring, and MRR prediction, thereby restricting the transition of BM from empirical regulation to model-driven development.

## Comparison with other machining technologies

BM differs from mechanical machining, chemical etching, laser machining, electrical discharge machining, and electrochemical machining in material removal mechanism, energy input, surface integrity, and environmental impact. It removes materials primarily via microbial metabolites, redox reactions, or extracellular electron transfer, thereby offering low mechanical loading and limited thermal effects. However, its low MRR, insufficient dimensional accuracy, process instability, and limited industrial maturity remain major constraints. Therefore, its technical value should be evaluated using quantitative indicators such as MRR, SR, dimensional accuracy, energy consumption, cost, and environmental impact, as summarized in Table 4.

**Table 4:** Comparison between biomachining and other machining technologies

Machining technology	Removal mechanism	MRR	Surfacequality	Energy andcost	Environmental issues	Applicability	References
Conventional machining	Mechanical cutting or abrasion	High; suitable for bulk removal	High; suitable for bulk removal	Mature equipment; energy depends on the machine and load	Cutting fluid, chips, and tool wear	Bulk removal, high-efficiency manufacturing	Li et al. (2025)
Chemical etching	Chemical dissolution	Moderate to high; dependent on etchant and material	Good patterning; possible over-etching and undercutting	Low equipment cost; chemical and waste-treatment costs	Strong acids/alkalis, toxic etchants, and waste liquid	Pattern transfer, thin films, and microstructures	Kohn et al. (2024); Hocheng and Jadhav (2015)
Laser machining	Thermal melting or vaporization	High local removal rate	High precision; possible heat-affected zone, recast layer, and microcracks	High equipment cost and energy input	Fume, particles, and thermal effects	Microholes, grooves, and complex structures	Lesi et al. (2025)
Electrical Discharge Machining (EDM)	Thermal erosion by discharge	Moderate to high for conductive materials	Good shape capability; possible recast layer and thermal damage	High equipment and dielectric management costs	Dielectric fluid, debris, and thermal damage	Hard conductive materials and microfeatures	Zhang et al. (2018)
Electrochemical Machining (ECM)	Electrochemical dissolution	High for conductive materials	No tool wear and low mechanical damage	No tool wear and low mechanical damage	No tool wear and low mechanical damage	Complex conductive parts and difficult-to-cut metals	Maharana et al. (2020)
Biomachining (BM)	Microbial metabolism and bioelectrochemical dissolution	Generally low; long processing time	Low thermal/mechanical damage; limited accuracy and repeatability	Low external energy input; culture and sterilization costs	Waste medium, metal ions, and biosafety concerns	Low-damage microstructures and functional surfaces	Pradeep et al. (2022)
Hybrid biomachining (HBM)	Microbial dissolution assisted by external fields or auxiliary processes	Potentially higher than BM	Possible MRR improvement	Additional equipment and control costs	Combined biological, physical, and chemical waste streams	Process intensification and controlled biomachining	Song et al. (2025); Uno et al. (1996)

Compiled by the authors based on the cited literature

Compared with conventional mechanical machining such as milling, grinding, and drilling, BM does not rely on strong tool-workpiece contact, thereby reducing mechanical loading, residual stress, and subsurface damage (Li et al. 2025). This makes it more suitable for thin-walled structures, brittle material surfaces, and low-damage microstructural fabrication (Taufiqurrakhman et al. 2021). However, conventional machining still has clear advantages in MRR, machining accuracy, equipment maturity, and batch stability. Because BM typically has a low MRR and long processing time, it is currently better suited to localized fine removal, surface functionalization, and tool dressing rather than high-efficiency bulk material removal.

Compared with chemical etching, BM is also a dissolution-based process, but it uses milder microbial metabolites or biological oxidation cycles instead of strong acids, alkalis, or oxidizing etchants. This can reduce risks associated with over-etching, safety hazards, and corrosive waste (Hocheng et al. 2012b ; Li et al. 2000). However, the environmental advantage of BM should not be assumed solely from its mild reaction conditions (Kohn et al. 2024; Hocheng and Jadhav 2011). It should be evaluated against competing processes under the same functional unit, considering energy consumption, chemical use, waste treatment, emissions, cost, machining quality, and process efficiency.

Compared with laser machining and electrical discharge machining, BM produces almost no heat-affected zone, recast layer, or thermal cracks, thereby preserving surface integrity. Laser machining and electrical discharge machining are efficient and controllable for microholes, microgrooves, and complex structures. However, their high local energy input can lead to surface alterations, microcracks, residual thermal stresses, high equipment costs, and increased energy consumption (Lesi et al. 2025). BM is more suitable for heat-sensitive materials, high-integrity surfaces, and functional microstructures. However, its low MRR, limited spatial selectivity, and slow process response still restrict its use in high aspect ratio structures, highly uniform arrays, and rapid manufacturing (Matlani and Kadam 2021; Díaz-Tena et al. 2014).

Compared with electrochemical machining, BM also involves interfacial electrochemical dissolution, but differs in driving force and control mode. Electrochemical machining uses an external electric field and electrolyte circulation to achieve high MRR and complex-shape machining. Still, it requires precise control of the machining gap, electrolyte state, and power parameters, with additional energy, electrolyte treatment, and equipment costs (Zhang et al. 2018). BM is mainly driven by microbial metabolism and redox cycles, which may reduce external energy input, but its reaction rate, stability, and controllability remain limited. Therefore, BM is more suitable as a complementary process for small-scale, low-damage applications than as a short-term replacement for electrochemical machining.

HBM offers a potential route to improve MRR and process controllability. External assistance, including electric fields, flow fields, jets, magnetic fields, and ultrasound, can enhance mass transfer, oxidant regeneration, and interfacial reactions. However, its effectiveness should not be judged solely by MRR improvement, as external fields may also affect microbial activity, metabolic stability, SR, and undercutting (Song et al. 2025; Uno et al. 1996). Therefore, the key issue is to clarify the relationships among external field parameters, microbial metabolism, interfacial reactions, and machining quality, rather than treating HBM as a universal solution.

Overall, BM is better suited to fine processing with low mechanical loading, limited thermal damage, and potential environmental benefits than to high efficiency bulk removal. Its promising applications include low-damage microstructures, functional surfaces, tool dressing, and biocompatible surface modification. However, limitations in MRR, dimensional accuracy, repeatability, equipment maturity, and verification of environmental impact remain. Therefore, the practical value of BM should be assessed against competing machining technologies under the same functional unit, considering energy consumption, cost, waste treatment, environmental impact, and machining performance.

## Biomachining processes and technologies

BM processes can be classified into conventional BM and HBM according to the use of external assistance. Conventional BM relies on microbial metabolism and bioelectrochemical reactions to remove material. In contrast, HBM introduces external fields or auxiliary methods to enhance mass transfer, oxidant regeneration, and process controllability. Based on the mechanisms discussed above, this section reviews conventional BM, HBM, and related process modeling and optimization strategies, aiming to clarify the current technological framework and development direction of BM processes.

### Conventional biomachining

Selective material removal is essential for applying BM to microfabrication. In most cases, BM is combined with masking techniques to confine the contact between microbial metabolites and the material surface, thereby enabling the fabrication of microgrooves, micro-pits, and complex patterns (Song et al. 2025; Muhammad et al. 2018; Díaz-Tena et al. 2014). Masking approaches such as digital lithography, maskless lithography, and laser ablation of resists have been used to prepare mask patterns for biomachined microstructures, thereby promoting their application in micromachining (Imran et al. 2017; Suwandi et al. 2014; Saragih and Ko 2013).

**Fig. 5:**
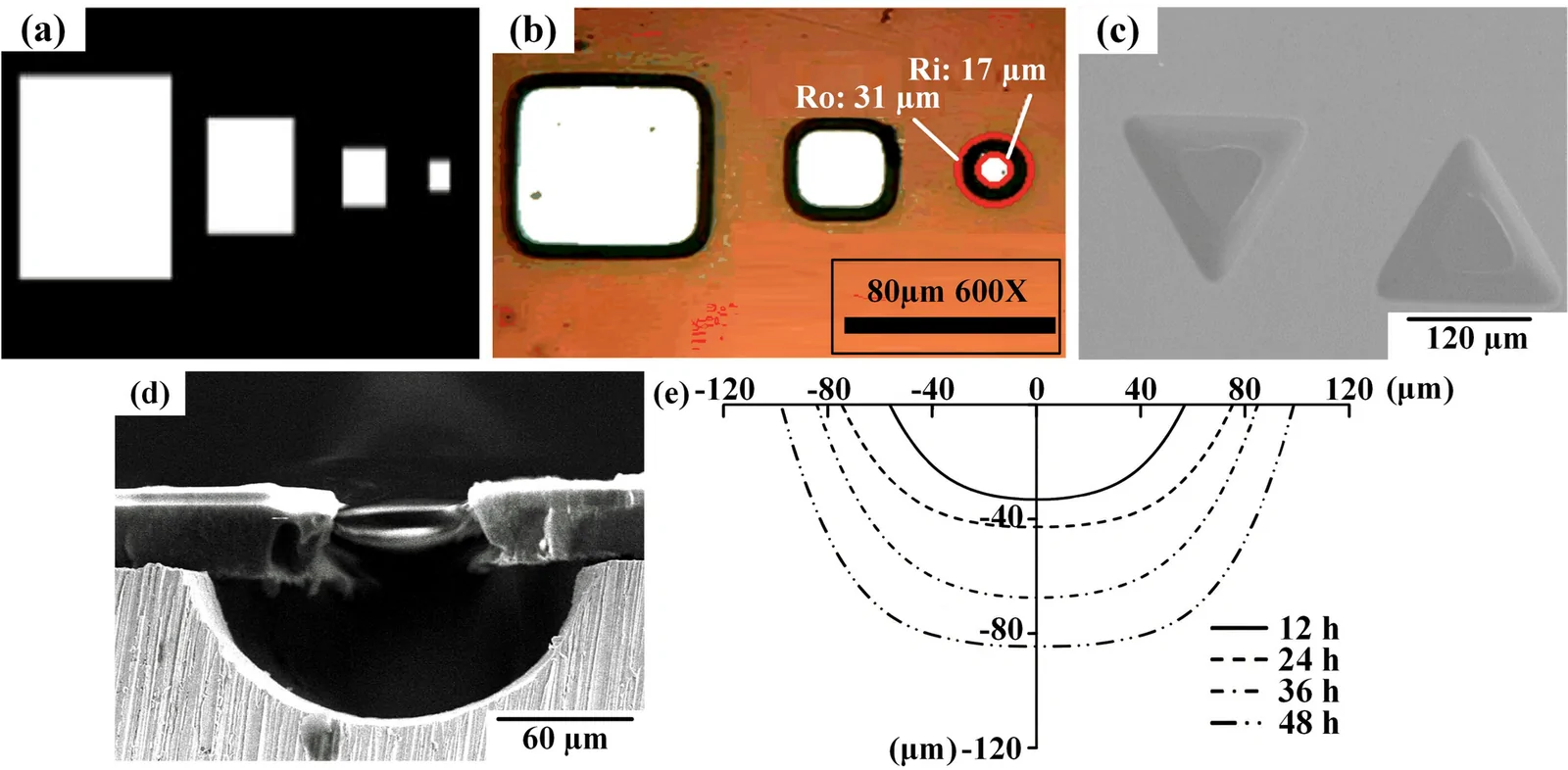
Mask dimensional effect and microstructure formation characteristics in biomachining. **a** Designed and **b** fabricated rectangular mask; **c** SEM image of the fabricated microstructure; **d** undercutting and **e** morphological evolution of microstructures during biomachining. Panel sources and permissions: **a**–**c** adapted from Figs. 6 and 11d, respectively, of Imran et al. (2017), with permission from Springer Nature Customer Service Centre GmbH. This material is not covered by the Creative Commons license of this article. **d** and **e** reprinted from Figs. 4 and 9, respectively, of Istiyanto et al. (2011), Copyright (2011), with permission from Elsevier

However, the accuracy of the mask pattern does not directly determine the final machining accuracy, as shown in Fig. 5a–c. Rounded photoresist edges, nonuniform mask adhesion, and pattern transfer errors can alter the initial machining boundary (Imran et al. 2017). In addition, metabolite diffusion, local pH and ORP fluctuations, and reaction product layer formation during BM can further cause the actual removal region to deviate from the designed pattern. Therefore, dimensional accuracy is governed not only by mask quality, but also by biochemical reactions and interfacial mass transfer.

Existing studies have shown that BM can fabricate microscale structures such as microgrooves, micro-pits, and complex surface patterns, as summarized in Table 5, thereby indicating its potential for low-damage microfabrication. However, shape errors and dimensional deviations remain common (Istiyanto et al. 2017, 2012; Muhammad et al. 2015; Hocheng et al. 2012c). Undercutting is one of the main causes of dimensional error, as shown in Fig. 5d and e. It results from the lateral diffusion of oxidative metabolites and ions near the mask edge, as well as the quasi-isotropic corrosion behavior of BM (Istiyanto et al. 2011). Longer processing times usually intensify undercutting, leading to lower aspect ratios, rounded edges, and reduced pattern fidelity (Singh et al. 2019). Therefore, undercutting control is essential for transforming BM from feasible microstructure fabrication into precise microfabrication.

**Table 5:** Summary of microstructure characteristics in biomachining

Material	Feature type	Time (h)	Feature size/depth	Key finding	Main limitation	References
Cu, Ni, Al	Lines, circles, squares	48	70 μm features; aspect ratios: 0.61, 0.42, 0.77	Aspect ratio varied with feature type and material	Aspect ratio decreased with increasing line width	Hocheng et al. (2012b)
Cu	Grooves, pits, complex patterns	12	110 μm grooves; 245 μm pits; 3 μm minimum grooves	Various microfeatures were fabricated	Lateral machining rate varied from 1.0 to 3.2 μm/h	Istiyanto et al. (2012)
Cu	Lines, squares, circular pits	1.7	35 μm lines; 50–60 μm squares; 30 μm pits	High feature density reached 140 pits/mm^2^	MRR decreased with time	Imran et al. (2017)
Cu	Grooves	24	Depth increased from 24.4 to 43.1 μm	Longer time increased the groove depth	SR increased from 1.5 to 2.0 μm	Istiyanto et al. (2017)
Cu	Grooves	48	Width increased from 50 to nearly 200 μm; depth about 80 μm	Unequal lateral and vertical etching	Undercutting was significant	Istiyanto et al. (2011)
Cu	Grooves	32	About 300 μm lines	Machining time affected the workpiece profile	Undercutting occurred	Singh et al. (2019)

All listed cases used *Acidithiobacillus ferrooxidans*. The reported dimensions and performance values were obtained under different experimental conditions and are provided as representative results rather than for direct quantitative comparison

Compiled by the authors based on the cited literature

In addition, the repeatability of biomachined microstructure fabrication has not been sufficiently validated. Fluctuations in microbial activity, medium composition, pH, ORP, OCP, metabolite concentration, surface oxide films, and mask adhesion can cause variations in MRR, machining depth, lateral corrosion, and surface quality. However, most studies still report representative morphologies or single experimental cases, with limited statistical evaluation of dimensional deviation, undercutting, morphology consistency, and surface integrity. The key limitation is the lack of standardized evaluation metrics for accuracy and repeatability, which hinders the transition of BM from feasible microstructure fabrication to controllable microfabrication.

### Hybrid biomachining

HBM introduces external fields, auxiliary processes, or other machining methods to address the low MRR, limited mass transfer, and insufficient controllability of conventional BM (Chavoshi and Luo 2015). It generally includes two strategies: coupling BM with external fields, such as electric fields, flow fields, and jets, to enhance interfacial reactions or mass transport; and using galvanic coupling, acid assistance, or other methods to modify the electrochemical environment and promote selective dissolution. However, current studies remain limited to a few materials and assistance methods. Therefore, the role of HBM should be evaluated by both removal efficiency and machining quality.

**Fig. 6:**
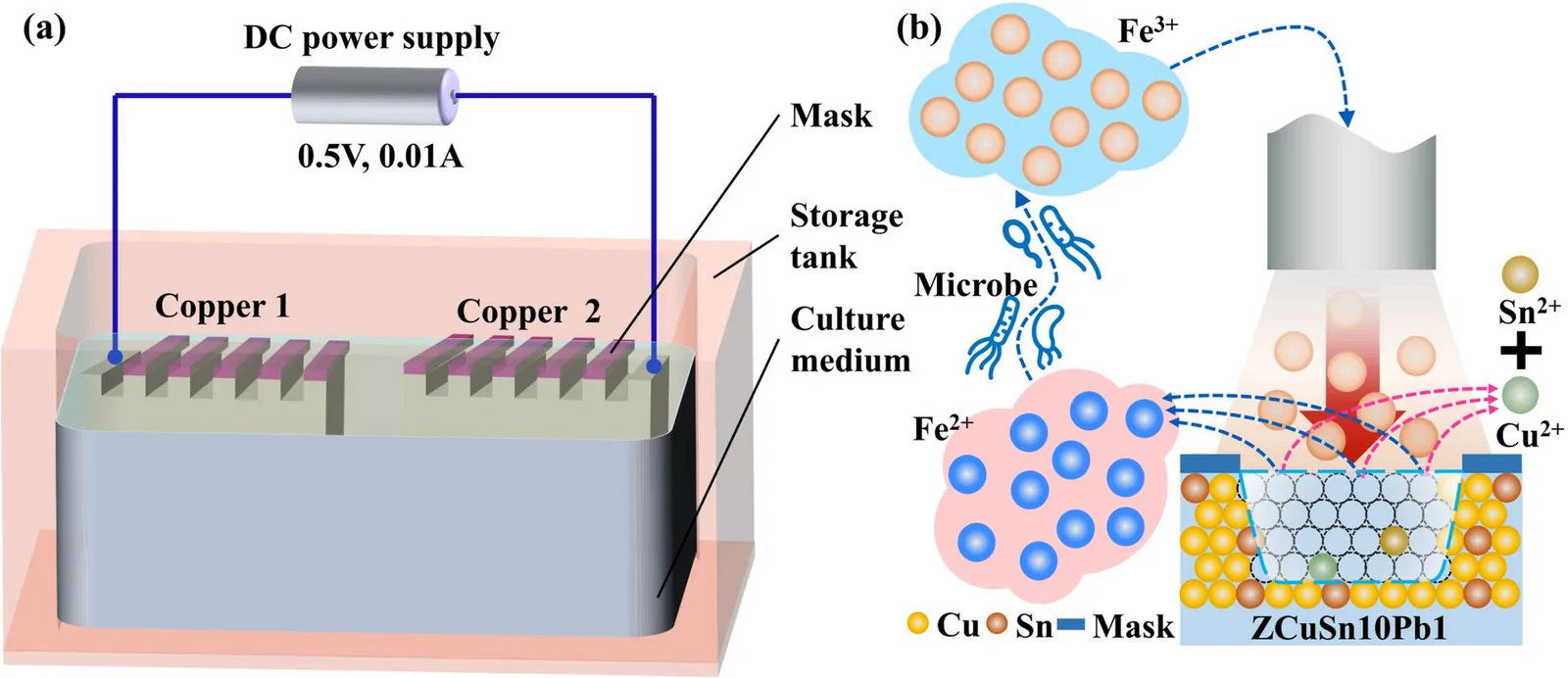
Schematic diagram of hybrid biomachining. **a** electric-field-assisted biomachining; **b** jet-assisted biomachining. Panel sources and permissions: **a** drawn by the authors based on Uno et al. (1996); **b** reprinted from Fig. 5b of Song et al. (2025), Copyright (2025), with permission from Elsevier

Existing studies indicate that external assistance can improve material removal capability to some extent, as shown in Table 6. Electric-field assistance can promote anodic dissolution by altering the workpiece potential. As shown in Fig. 6, Uno et al. reported that the removal depth of copper increased by approximately 52% after applying a voltage (Uno et al. 1996). Flow or jet assistance can enhance mass transfer between microbial metabolites and the workpiece surface, thereby increasing MRR (Song et al. 2025). In addition, Galvanic coupling uses the potential difference between different materials to strengthen selective dissolution in the anodic region (Ma et al. 2020b ). Acid assistance can reduce interfacial reaction resistance by increasing the H^+^ concentration, thereby promoting metal dissolution (Sonia et al. 2021). These studies suggest that hybrid strategies have the potential to intensify BM processes.

**Table 6:** Comparison of hybrid biomachining strategies based on removal efficiency and surface quality

Hybrid strategy	Material	MRR	SR	Critical comment	References
Electric-field-assisted biomachining	Cu	15 → 24.5 μm/h	Not clearly quantified	Demonstrates electrochemical enhancement, but dimensional accuracy and microbial viability were not fully evaluated	Uno et al. (1996)
Jet-assisted biomachining	Cu, Fe, Co	Fe: 35 → 225 mg/ (h∙cm^2^)	1.8 → 18.5 μm	Higher removal efficiency may sacrifice surface quality	Lin et al. (2024)
Jet-assisted microstructure fabrication	Cu alloys	1.4 → 6.9 mm^3^/h	1.2–4 μm	A balance between feature formation and surface integrity is required	Song et al. (2025)
Galvanic-coupled biomachining	Cu-Co alloy	2.4 → 7.4 mg/ (h∙cm^2^)	Not clearly quantified	Improved MRR, but limited evaluation of surface quality, accuracy, and stability	Ma et al. (2020b)
Acid-assisted biomachining	Al alloy	0.08 → 0.24 mg/ (h∙cm^2^)	ΔSa: − 1.7 → − 3.0 μm	Acid assistance may reduce biological specificity and environmental benefits	Sonia et al. (2021)

Compiled by the authors based on the cited literature

BM, Biomachining; MRR, Material removal rate; SR, Surface roughness; Sa, Arithmetical mean height

It should be noted that an increase in the MRR does not necessarily improve machining quality (Lin et al. 2024). Stronger interfacial reactions and mass-transfer disturbances may increase SR, local over-corrosion, non-uniform product deposition, undercutting, or microstructural distortion. For example, some jet-assisted studies showed that SR and MRR may increase simultaneously, indicating that higher removal efficiency may be achieved at the expense of SR (Song et al. 2025). Therefore, HBM should be evaluated not only by MRR or removal depth but also by SR, dimensional error, undercutting, surface integrity, and batch repeatability.

Overall, HBM is a promising approach to overcoming the low efficiency of conventional BM, but its engineering feasibility has not been sufficiently verified. The main barriers include unclear removal mechanisms under multi-field coupling, limited understanding of the effects of external fields on microbial activity, the unresolved trade-off between MRR and SR, and immature equipment and control methods. Therefore, single-case demonstrations are insufficient, quantitative relationships among external fields, microbial metabolism, interfacial reactions, and machining quality are needed to support controllable and engineering-oriented applications.

### Process modeling and intelligent optimization

For BM, process modeling should link biological variables and process parameters with machining responses. This is important for understanding material removal and improving process control (Chavoshi and Luo 2015). Relevant inputs include microbial activity, cell density, growth phase, culture environment, metabolite concentration, solution state, processing time, and material properties. In contrast, outputs include MRR, SR, dimensional accuracy, surface integrity, and environmental performance. Therefore, modeling and parameter optimization can help identify key variables and support the transition from empirical regulation to predictable machining.

Existing studies have used significance analysis, sensitivity analysis, orthogonal experiments, Taguchi methods, response surface methodology, linear regression, and intelligent optimization algorithms to model or optimize the relationships between culture/process parameters and material removal characteristics (Pradeep et al. 2024; Ma and Huang 2020a; Singh et al. 2019), as shown in Table 7. These methods help shorten experimental cycles, identify key parameters, and obtain improved process conditions. For example, response surface methodology and orthogonal experiments are commonly used to analyze the effects of pH, temperature, culture time, Fe^2+^/Fe^3+^ concentration, and processing time on MRR and SR (Pradeep et al. 2022; Muhammad et al. 2015).

**Table 7:** Process modeling and optimization studies in biomachining

Microorganism	Material	Target	Method	Key findings	Optimal conditions	References
*Acidithiobacillus ferrooxidans*	Pure Fe	Maximize MRR	Taguchi method	Agitation speed was the most significant factor, followed by temperature, pH, and FeSO_4_ concentration	160 rpm, 45 °C, pH 2.6, 16 wt% FeSO_4_; MRR: 149.7 mg/h; depth: 24.1 μm	Muhammad et al. (2015)
*Acidithiobacillus ferrooxidans*	Cu	Predict MRR and SR	Response surface methodology	Empirical model for simultaneous MRR and SR prediction	170 rpm, 30 °C, pH 2.5, 16.6% FeSO_4_; MRR: 83.6 mg/h; SR: 0.2 μm	Muhammad et al. (2018)
*Acidithiobacillus ferrooxidans*	Cu	Maximize MRR	Response surface methodology	Machining time, inoculum size, and inoculum age were key factors	Inoculum concentration: 7.5 vol%; maximum MRA: 0.56 g	Singh et al. (2019)
*Acidithiobacillus ferrooxidans*	Al alloy	Maximize MRR; minimize SR	Taguchi method	MRR and SR are related to pH, agitation speed, time, and cell concentration	pH 2, 100 rpm, 24 h, 10^4^ CFU/mL; MRR: 0.26 mg/(h·cm^2^); SR: 41.4 μm	Sonia et al. (2021)
*Acidithiobacillus ferrooxidans*	Ni, Ti, NiTi	Maximize MRR; minimize SR	Taguchi method	Optimal conditions were material-dependent	30 °C; material-specific pH and ferric ion content; MRA within 72 h: 0.23—0.59 g	Pradeep et al. (2023)
*Paracoccus versutus*	NiTi	Maximize MRR; minimize SR	Grey wolf optimizer	Predictive model incorporating microbial and process parameters	MRR optimum: 95 rpm, 25 °C, pH 4.4; SR optimum: 80 rpm, 25 °C, pH 2.5	Pradeep et al. (2024)

Compiled by the authors based on the cited literature

MRR, Material removal rate; SR, Surface roughness; MRA, Material removal amount

However, current BM models remain largely based on empirical fitting and single-objective optimization, with limited predictive capability and engineering applicability (Pradeep et al. 2023). A major reason is that BM is governed not only by fixed process parameters, but also by dynamic biological variables. Microbial activity, cell density, growth phase, pH, ORP, DO, Fe^2+^/Fe^3+^ ratio, metabolite concentration, and biofilm state can directly affect material dissolution and machining stability. These variables are often time-varying and batch-dependent, making it difficult to define inputs consistently and transfer models.

In addition, most existing models focus mainly on MRR, while SR, dimensional accuracy, surface integrity, environmental impact, and cost are insufficiently considered. The lack of mechanistic constraints from microbial metabolism, electrochemical reactions, and mass transfer further limits model interpretability. These limitations become more significant in HBM, where external fields increase the complexity of parameter coupling and may also affect microbial activity.

Although direct applications of machine learning (ML) and Internet of Things (IoT) concepts in BM remain limited, related advances in optimization, bioleaching, and smart BM offer useful references for future process control (Shariatifar et al. 2025; Isoko et al. 2024; Naseri et al. 2023). To overcome the above limitations, BM modeling should move toward mechanism-data fusion frameworks that connect microbial metabolism, electrochemical reactions, mass transfer, and material dissolution with machining responses. Supported by online sensing of pH, oxidation–reduction potential, temperature, DO, ion concentration, and microbial activity, ML models can help describe nonlinear relationships among microbial state, culture environment, process parameters, and machining responses, such as MRR, SR, dimensional error, and surface integrity (Mondal et al. 2023). Further integration with digital twin-based closed-loop control may support anomaly detection, prediction of removal, and real-time process regulation. It may also enable multi-objective optimization of MRR, SR, dimensional accuracy, and environmental performance.

## Applications of biomachining in manufacturing

BM uses microorganisms and their metabolites to remove materials and has attracted attention in microfabrication, surface engineering, and functional device fabrication due to its low mechanical loading, limited thermal damage, and potential green manufacturing characteristics (Hocheng et al. 2012a). However, most existing applications are still at the stage of laboratory validation or prototype fabrication, while cases for continuous industrial production remain limited. Therefore, as summarized in Table 8, this section reviews current applications in microstructure fabrication, functional surfaces, microchannels, tool dressing, and functional device fabrication, while distinguishing their laboratory demonstration value from their potential limitations for industrial application.

**Table 8:** Representative applications, industrial relevance, and limitations of biomachining

Application area	Laboratory examples	Potential industrial relevance	Current limitations	References
Micro-components and microstructures	Micro-gears, grooves, pits, pillars, custom patterns	MEMS, sensors, microdevices	Low MRR; undercutting; dimensional error; poor repeatability	Liu et al. (2009); Zhang and Li (1998)
Functional surfaces	Tribological textures, wettability control, and cell adhesion surfaces	Lubrication control, anti-friction surfaces	Limited durability tests; insufficient batch evaluation	Liu et al. (2026b ); Whulanza et al. (2016)
Micro-channel fabrication	Polydimethylsiloxane molds, microchannel heat sinks	Microfluidics, heat exchangers	Limited scale; uncertain accuracy and long-term performance	Santaolalla et al. (2023)
Tool dressing	Metal-bond grinding plates, diamond wheels	Grinding tool conditioning	Dressing rate control; process stability; integration with grinding systems	Lin et al. (2026); Ma and Huang (2020b)
Functional devices	Microfluidic chips, microbial fuel cell electrodes	Energy devices, biosensors	limited device-level validation and long-term stability	Suwandi et al. (2014); Bijimol et al. (2025)

Compiled by the authors based on the cited literature

### Machining of micro-components and microstructures

**Fig. 7:**
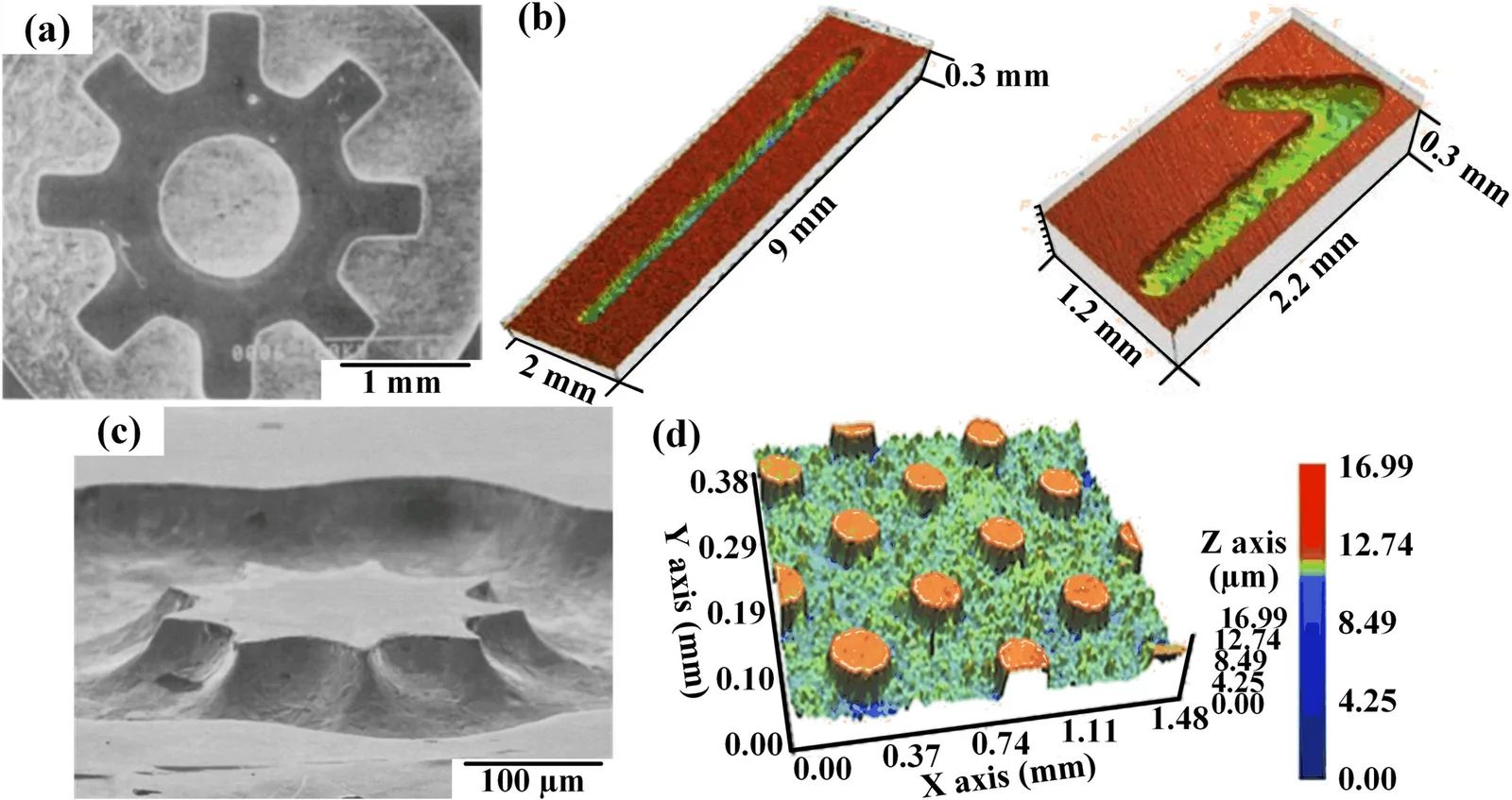
Applications of biomachining in micromachining. **a** Micro-gear; **b** complex-shaped feature; **c** complex microstructure patterns and **d** micropillars. Panel sources and permissions: **a** adapted from Fig. 4a of Zhang and Li (1998), with permission from Springer Nature Customer Service Centre GmbH. This material is not covered by the Creative Commons license of this article. **b** reprinted from Fig. 14 of Díaz-Tena et al. (2014), Copyright (2014), with permission from Elsevier. **c** reprinted from Fig. 7b of Istiyanto et al. (2012), Copyright (2012), with permission from Elsevier. **d** adapted from Fig. 9b of Muhammad et al. (2015), with permission from Springer Nature Customer Service Centre GmbH. This material is not covered by the Creative Commons license of this article

Micro-component and microstructure fabrication is one of the earliest application areas of BM. As shown in Fig. 7, structures such as micro-gears, microgrooves, micro-pits, and complex patterns have been fabricated, indicating the feasibility of BM for low-damage microstructure formation (Muhammad et al. 2018, 2015; Díaz-Tena et al. 2014; Zhang and Li 1998). However, these studies remain mainly at the laboratory-scale validation stage, and there is still a clear gap to industrial microstructure manufacturing. Key limitations include long processing time, low MRR, obvious undercutting, insufficient dimensional consistency, and limited repeatability verification (Istiyanto et al. 2012). Therefore, the key issue is no longer only whether microstructures can be fabricated by BM, but whether they can be fabricated stably, accurately, and repeatably.

### Preparation of functional surfaces

**Fig. 8:**
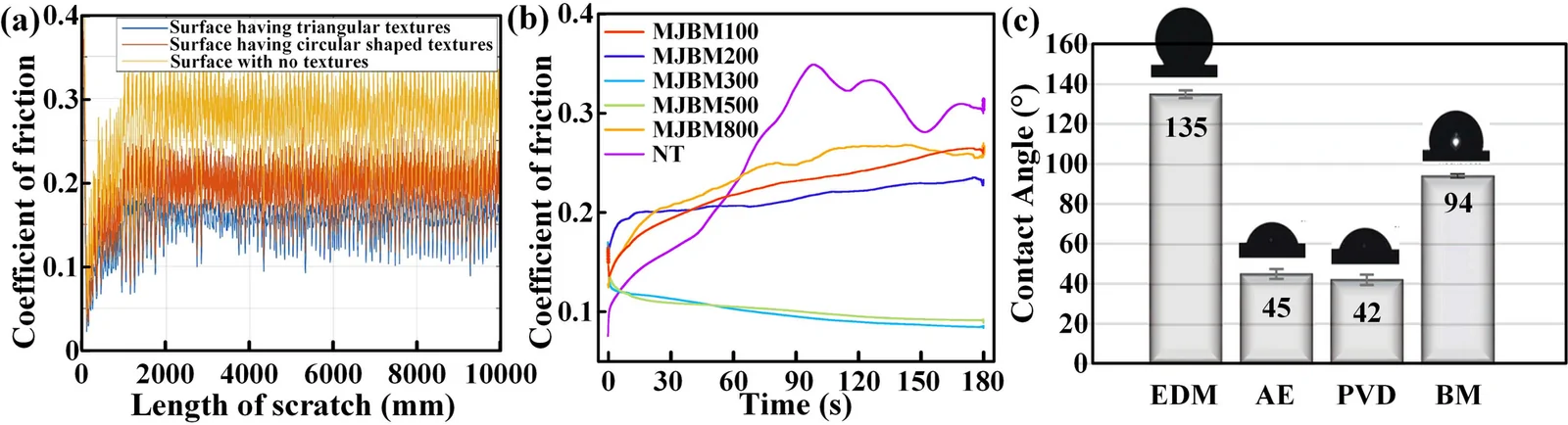
Applications of biomachining in functional surface modification. **a** Friction coefficient curves of different texture patterns; **b** friction coefficient curves of different feature sizes; **c** contact angles of implant surfaces after different treatments. Panel sources and permissions: **a** adapted from Fig. 20 of Imran et al. (2017), with permission from Springer Nature Customer Service Centre GmbH. This material is not covered by the Creative Commons license of this article. **b** reprinted from Fig. 3c of Song et al. (2025), Copyright (2025), with permission from Elsevier. **c** reprinted from Fig. 1b of Qosim et al. (2018), with the permission of AIP Publishing

Functional surface fabrication is a promising application of BM. By controlling surface microstructures and morphology, BM can improve tribological, wetting, and interface properties (Liu et al. 2026b; Song et al. 2025; Imran et al. 2017). As shown in Fig. 8, it has been used to fabricate friction-reducing textures, hydrophobic surfaces, and cell-adhesive structures (Qosim et al. 2018; Whulanza et al. 2016). However, most studies remain limited to laboratory validation on specific samples and mainly correlate biomachined surface morphology with short-term functional performance. The combined effects of surface chemistry, oxide layers, reaction residues, and service conditions are rarely evaluated. Therefore, evaluation criteria should move beyond single-sample functional testing and include large-area uniformity, durability, batch repeatability, and long-term performance stability.

### Micro-channel fabrication

**Fig. 9:**
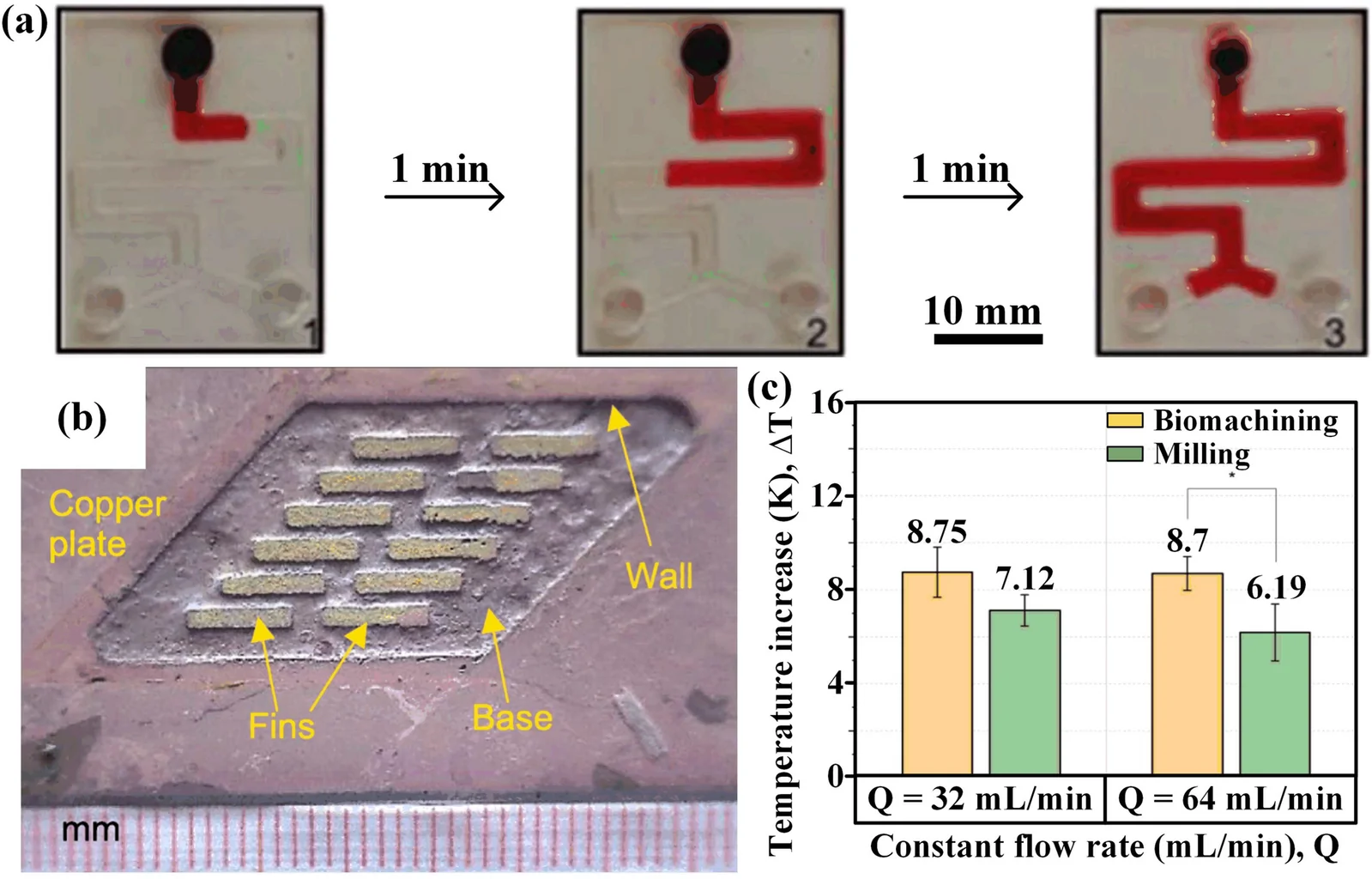
Applications of biomachining in microchannel fabrication. **a** Self-powered polydimethylsiloxane microfluidic device; **b** fabricated microchannel heat sink and **c** temperature drop for microchannel heat sinks fabricated by BM and milling. Panel sources and permissions: **a** reprinted from Fig. 5b of Santaolalla et al. (2023), Copyright (2023), with permission from Elsevier. **b** and **c** reprinted from Figs. 5c and 11, respectively, of Taufiqurrakhman et al. (2021), Copyright (2021), with permission from Elsevier

Microchannel structures are important for fluid transport, mixing, and heat transfer in microfluidic devices, cooling structures, and biochemical detection systems. As shown in Fig. 9, BM has been used to fabricate microchannel molds and prototype devices, such as polydimethylsiloxane microfluidic devices replicated from metal molds for directional droplet motion control (Santaolalla et al. 2023). However, current studies mainly remain at the stage of mold fabrication and prototype validation (Taufiqurrakhman et al. 2021). Since microchannels require high dimensional accuracy, controlled cross-sectional profiles, clean surfaces, and consistent flow resistance, long processing time, lateral removal, and reaction residues may affect channel accuracy and device performance. Therefore, the key issue is whether BM-fabricated microchannels can meet device-level requirements for cross-sectional accuracy, surface cleanliness, flow resistance, and long-term stability.

### Tool dressing

**Fig. 10:**
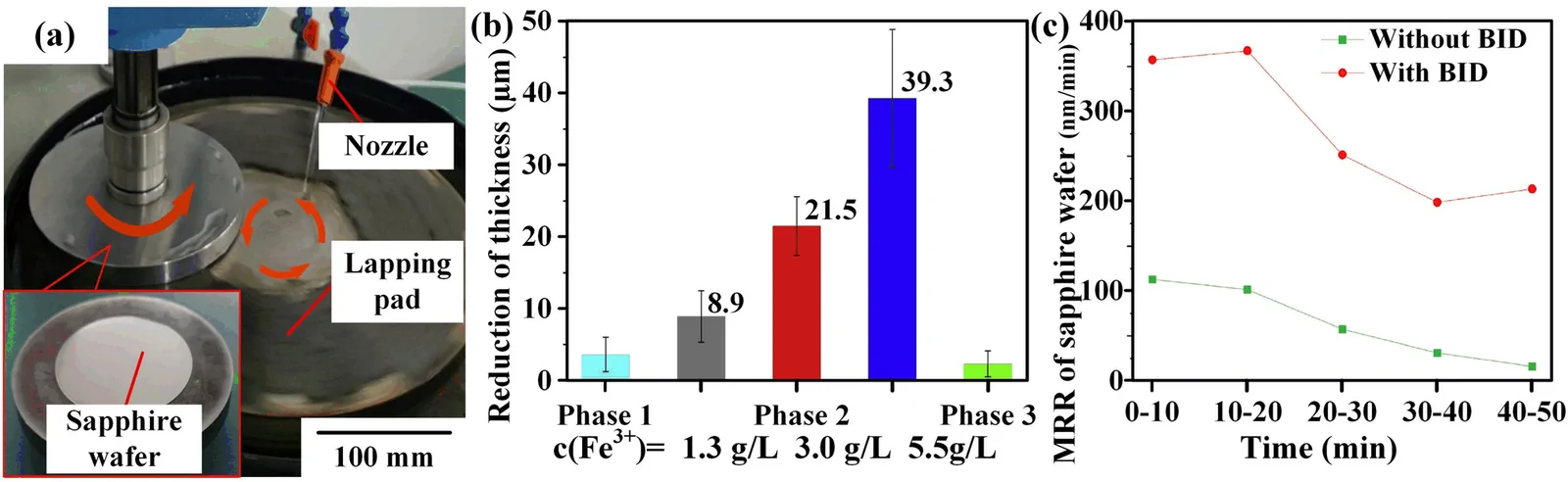
Applications of biological in-process dressing (BID) in abrasive tool conditioning. **a** BID of a diamond grinding tool for sapphire wafer grinding; **b** improvement in the MRR of lapping pads by BID; **c** comparison of sapphire grinding with and without BID. Panel sources and permissions: **a** and **c** reprinted from Figs. 4 and 5, respectively, of Ma et al. (2025a), Copyright (2025), with permission from Elsevier. **b** reprinted from Fig. 11 of Ma and Huang (2020b), Copyright (2020), with permission from Elsevier

Tool dressing is one of the BM applications closest to engineering use. Unlike direct microstructure fabrication, it aims to selectively remove metal bond or loading layers rather than precisely replicate microscale geometries, thereby improving abrasive protrusion, chip space, and cutting ability (Lin et al. 2026). As shown in Fig. 10, BM has been used to dress fixed abrasive lapping plates and metal-bonded diamond grinding wheels, thereby improving subsequent grinding performance (Ma et al. 2025a; Ma and Huang 2020b). However, dressing rate, uniformity, abrasive retention, and online integration remain key challenges. Insufficient dressing may leave residual bond or loading layers, while excessive dressing may weaken abrasive retention and shorten tool life. Therefore, BM-based dressing should be evaluated based on abrasive protrusion, grinding force, grinding temperature, SR, and tool life, rather than on bond removal efficiency alone.

### Functional device fabrication

**Fig. 11:**
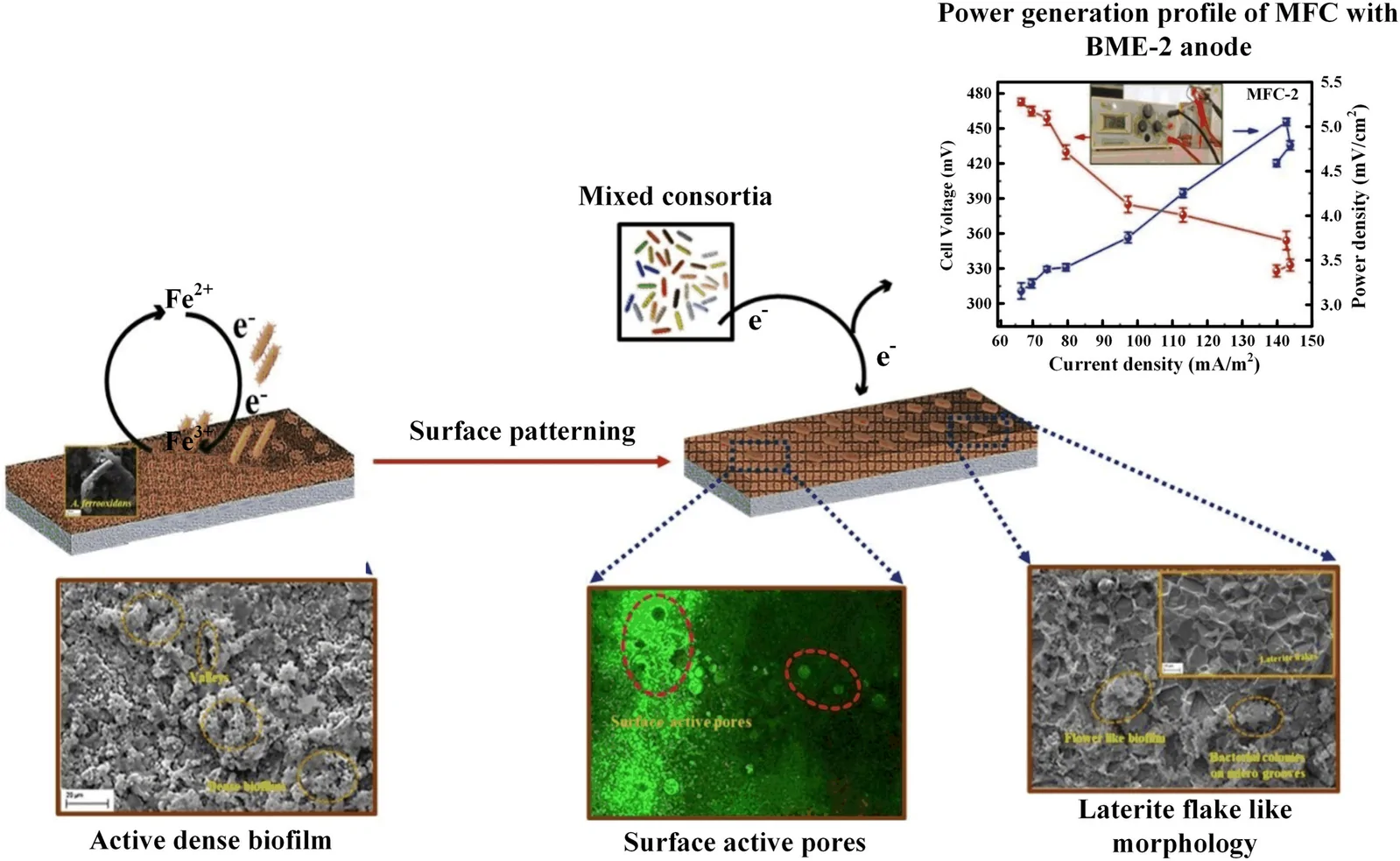
Applications of biomachining in microbial fuel cell fabrication Source and permission: Reprinted from Fig. 5 of Bijimol et al. (2025), Copyright (2025), with permission from Elsevier

BM has also been used for structural regulation and interface modification of functional devices. As shown in Fig. 11, it has been applied to microfluidic chips, electrode structures, and microbial fuel cells (Suwandi et al. 2014). For example, porous anodes can increase specific surface area and enhance bacteria-electrode interactions and electron transfer, thereby improving microbial fuel cell performance (Bijimol et al. 2025). However, most applications remain at the proof-of-concept or prototype stage. Functional device fabrication requires structural accuracy, interface cleanliness, device consistency, and long-term stability. Some microstructures may enhance interfacial reactions but reduce substrate mechanical strength (Hemalatha et al. 2021). Therefore, the critical issue is determining whether BM-induced interface structures can improve device performance without sacrificing structural accuracy, interface cleanliness, substrate strength, or long-term stability.

### Summary of application maturity and future requirements

Overall, BM has demonstrated potential in microstructure fabrication, functional surfaces, microchannels, tool dressing, and functional device modification. However, aside from tool dressing and some surface-modification applications, most studies remain at the prototype stage and have not yet established stable, repeatable, and scalable industrial processes. These limitations indicate that the main bottleneck is the lack of a stable and scalable process chain rather than the absence of isolated application demonstrations. Key issues include machining efficiency, dimensional accuracy, batch repeatability, and long-term performance, as well as equipment integration, contamination control, microbial stability, medium circulation, waste treatment, automation, inline monitoring, and process standardization.

## Challenges, scale-up barriers, and future perspectives

**Fig. 12:**
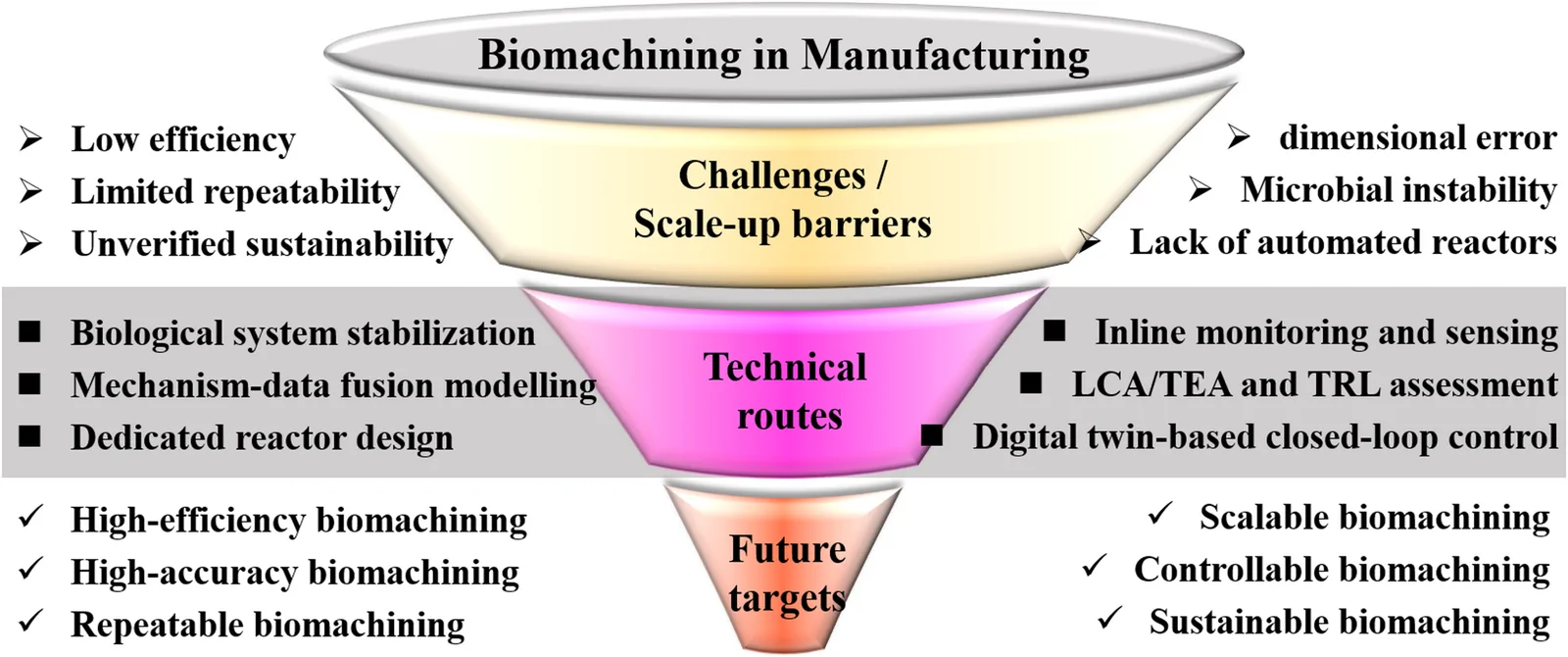
Key challenges, technical routes, and future targets for biomachining scale-up. Created by the authors

Although BM has been demonstrated in various laboratory applications, including functional surfaces, tool dressing, and functional device modification. However, its transition toward engineering, continuous, and scalable manufacturing still faces significant barriers. Unlike conventional manufacturing processes, BM is governed by the coupled effects of microbial metabolism, electrochemical reactions, interfacial mass transfer, and material dissolution, making the process highly dynamic and uncertain. Therefore, building on the application maturity analysis above, this section summarizes the key barriers to scale-up and proposes future routes toward a controllable, engineering-oriented BM, as shown in Fig. 12.

### Key challenges and scale-up barriers

At present, the transition of BM from laboratory validation to industrial application faces several major challenges.

First, machining efficiency and forming accuracy remain major technical constraints. The relatively low MRR leads to long processing times, which may aggravate undercutting, increase SR, and cause dimensional deviations. In addition, reaction product layers, passive films, and metabolite concentration gradients can lead to non-uniform material removal, making it difficult to control microstructural profiles, aspect ratios, and surface integrity. Therefore, improving removal efficiency must be balanced with dimensional accuracy and surface quality.

Second, microbial stability is a key bottleneck for scalable BM. During long-term cultivation or repeated processing, genetic instability, mutation, metabolic variability, biofilm evolution, microbial competition, contamination, nutrient depletion, and metal ion accumulation can change microbial activity and metabolite production. These changes may lead to fluctuations in MRR, SR, and machining repeatability. Therefore, scalable BM requires long-term culture maintenance, stable strain management, contamination control, medium renewal, microbial activity monitoring, and regulation of key solution parameters, including pH, ORP, DO, nutrient concentration, Fe^2+^/Fe^3+^ ratio, and metal ion concentration.

Third, manufacturing-oriented equipment and process integration remain insufficient. Most current studies still rely on beakers, culture dishes, or simple flow cells, which are difficult to connect with stable production lines. Dedicated reactor or machining-cell systems are needed to integrate microbial cultivation, machining reaction, medium circulation, temperature control, pH/ORP regulation, DO supply, waste treatment, metal ion recovery, and online monitoring. Continuous processing further requires stable microbial activity, nutrient supply, metabolite regulation, contamination prevention, and reaction product removal. In addition, inline monitoring of pH, ORP, DO, temperature, ion concentration, cell density, and machining depth is necessary for process diagnosis and closed-loop control. Process standardization, biosafety management, waste discharge control, regulatory compliance, economic feasibility, and technology readiness should also be evaluated before industrial implementation.

Finally, the sustainability and economic feasibility of BM still require systematic verification. Its mild reaction conditions, low mechanical loading, and limited thermal damage should not be directly equated with green manufacturing advantages. The actual environmental performance depends on the whole process chain, including medium preparation, sterilization, microbial cultivation, processing time, waste treatment, metal ion recovery, biosafety management, and equipment operation. Therefore, Life Cycle Assessment (LCA) and techno-economic analysis are needed to clarify the real environmental and economic benefits of BM in specific application scenarios.

### Roadmap toward controllable and scalable biomachining

**Fig. 13:**
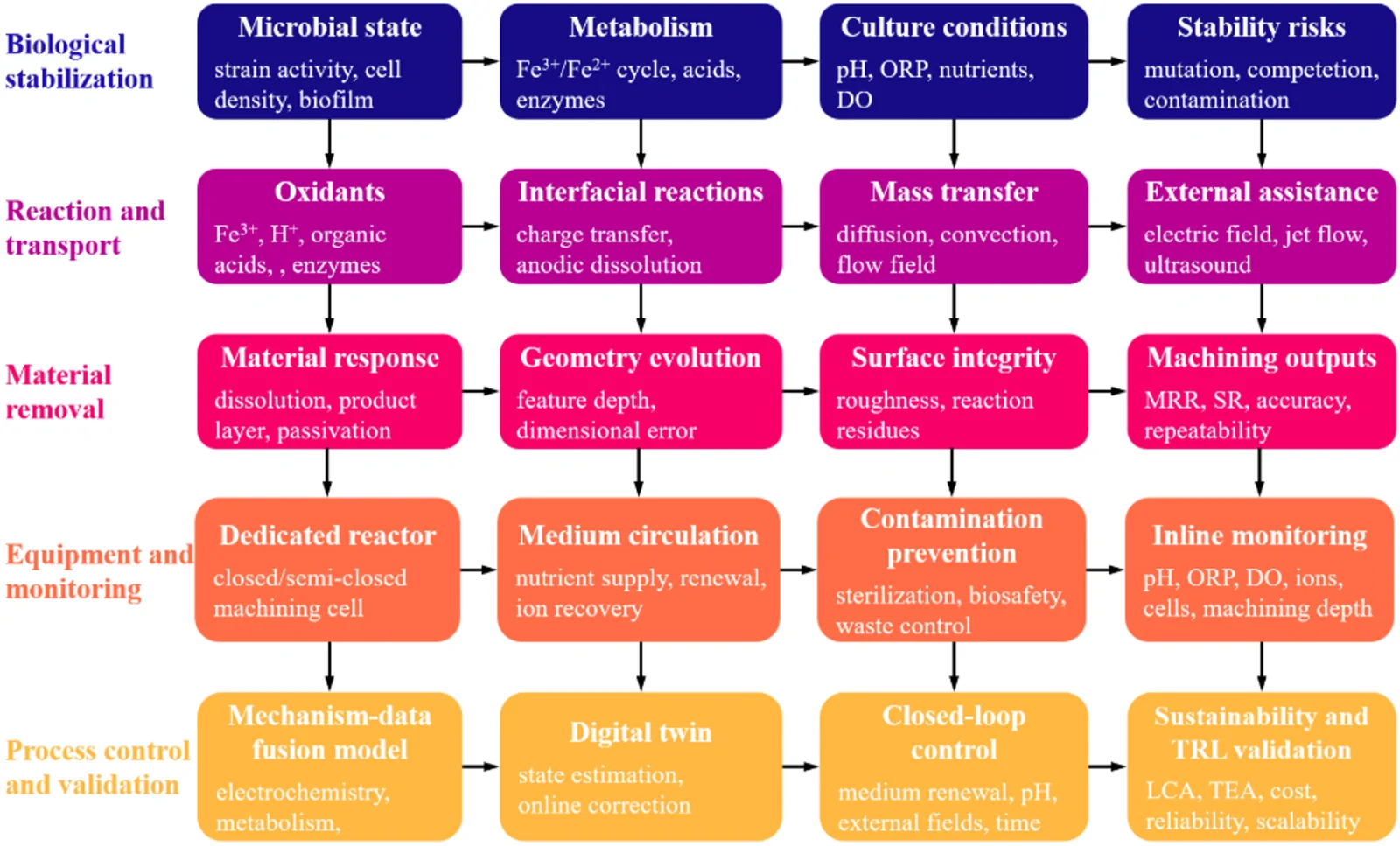
Integrated framework for controllable and scalable biomachining. Created by the authors

For engineering applications, BM should follow a route of biological system stabilization, mechanism-data fusion modeling, dedicated equipment development, digital twin-based closed-loop control, and sustainability validation, as shown in Fig. 13.

First, stable and repeatable microbial working windows should be established. For Fe^2+^/Fe^3+^ cycling systems, Fe^3+^ regeneration, pH, ORP, DO, and metal ion accumulation should be monitored. For organic acid or enzyme-mediated systems, metabolite production, medium stability, and product inhibition should be controlled. Strategies such as strain screening, strain preservation, immobilized cultivation, co-culture regulation, medium circulation, ion recovery, and online microbial monitoring can improve metabolic stability. Key indicators should include cell viability, metabolite concentration stability, pH/ORP fluctuation, continuous processing time, and MRR repeatability.

Second, predictive models that combine mechanistic constraints with data-driven methods should be developed. Kinetic models can describe charge transfer, diffusion resistance, and MRR, while data-driven models can map biological and process parameters to MRR, SR, dimensional error, and undercutting. However, future models should not only improve fitting accuracy but also explain the coupling among microbial metabolism, electrochemical reactions, mass transfer, and material dissolution. In addition, ML and IoT-based monitoring can provide useful support for this process. Online sensing of pH, ORP, temperature, DO, ion concentration, metabolite concentration, and microbial activity can be used to update ML models and digital twins. This enables prediction of removal, anomaly detection, and process-state estimation.

Third, dedicated BM equipment should be developed to enable stable, controllable, and scalable processing. A manufacturing-oriented BM system should evolve from simple laboratory containers to closed or semi-closed machining reactors integrating microbial cultivation, machining reaction, medium circulation, temperature control, pH/ORP regulation, DO supply, contamination prevention, sensor integration, waste treatment, and metal ion recovery. Continuous processing further requires stable microbial activity, nutrient supply, metabolite regulation, reaction product removal, and medium renewal over long operating periods. In addition, inline monitoring, process standardization, biosafety management, regulatory compliance, economic feasibility, and technology readiness should be evaluated before industrial implementation.

Fourth, digital twin-based closed-loop control should be introduced to link biological variability with manufacturing controllability. Real-time sensing data can provide dynamic process information, while physics-data fusion models can predict MRR, SR, dimensional error, undercutting, and process instability. Based on the target structure and quality requirements, key process variables such as medium renewal rate, flow rate, pH, nutrient supply, external-field parameters, and processing time can be adjusted to form a sensing-prediction-control-machining closed loop. Since microbial systems are inherently fluctuating, digital twin models should be updated online rather than relying on fixed parameters.

Finally, sustainability validation should be conducted using LCA and techno-economic analysis under clearly defined functional units, such as unit removal volume, unit machined area, or unit functional surface performance. The evaluation should consider energy consumption, medium and chemical use, sterilization, waste treatment, metal ion recovery, biosafety management, processing efficiency, equipment cost, and product quality. Such analysis can help identify application scenarios where BM offers real sustainability advantages.

### Milestone goals for engineering implementation

Based on the above roadmap, BM can be advanced through short-term, medium-term, and long-term goals. In the short term, the priority is to improve the repeatability of laboratory processes by standardizing strain preservation, culture medium formulation, pH/ORP monitoring, material pretreatment, MRR/SR evaluation, and repeatability metrics. In the medium term, predictable and controllable BM systems should be developed through mechanism-data fusion models, online monitoring, dedicated machining-cell design, and closed-loop regulation of microbial activity, metabolite concentration, flow conditions, and machining time. In the long term, engineering validation should be conducted for specific application scenarios, such as large-area functional surface treatment, microfluidic mold fabrication, and electrode surface modification. The evaluation should emphasize continuous operation, batch repeatability, lifetime performance, equipment reliability, waste treatment, cost accounting, and regulatory compliance.

Overall, the future development of BM should not rely merely on introducing concepts such as microbial engineering, artificial intelligence, or hybrid energy fields. Instead, the key is to transform microbial reactions into manufacturing processes that can be monitored, modeled, controlled, and verified. Only by moving from laboratory feasibility demonstration to engineering process control can BM achieve sustainable, scalable, and industrial applications.

## Conclusions

BM should be regarded as a complementary bio-assisted process for specific low-damage and function-oriented manufacturing applications, rather than a universal substitute for conventional or non-conventional machining. The main contribution of this review is to establish an integrated, manufacturing-oriented framework that links microbial activity, electrochemical dissolution, mass transfer, material response, process modeling, and application maturity. This framework clarifies the key barriers to BM development, including low MRR, dimensional error, poor repeatability, microbial instability, weak process controllability, and insufficient industrial verification. Overall, the broader manufacturing value of BM depends on transforming microbial reactions into processes that can be monitored, modeled, controlled, and validated through standardized evaluation, mechanism-data fusion modeling, dedicated equipment, inline monitoring, digital twin-based control, and sustainability assessment.

## Supplementary Information

Below is the link to the electronic supplementary material.

Supplementary Material 1

## Data Availability

All data generated or analyzed during this study are included in this published article.

## Abbreviations

BM: Biomachining
DO: Dissolved oxygen
HBM: Hybrid biomachining
IoT: Internet of things
LCA: Life cycle assessment
MIC: Microbiologically influenced corrosion
ML: Machine learning
MRR: Material removal rate
OCP: Open circuit potential
ORP: Oxidation-reduction potential
rpm: Revolutions per minute
SCE: Saturated calomel electrode
SR: Surface roughessn
