# Supplementary material for: Biomachining in manufacturing: mechanisms, processes, applications and future perspectives
Source: Bioresour Bioprocess. 2026 Oct 13;13(1):149. doi: 10.1186/s40643-026-01143-z (PMC13658698; doi:10.1186/s40643-026-01143-z)
Supplement: Supplementary file 1 [file 40643_2026_1143_MOESM1_ESM.docx]

**Supplementary Materials**

**Fig. S1**: Systematic workflow of literature search and article selection for biomachining-related studies. Created by the authors.
